# SPECS: Integration of side-chain orientation and global distance-based measures for improved evaluation of protein structural models

**DOI:** 10.1371/journal.pone.0228245

**Published:** 2020-02-13

**Authors:** Rahul Alapati, Md. Hossain Shuvo, Debswapna Bhattacharya

**Affiliations:** 1 Department of Computer Science and Software Engineering, Auburn University, Auburn, Alabama, United States of America; 2 Department of Biological Sciences, Auburn University, Auburn, Alabama, United States of America; Universidade Nova de Lisboa Instituto de Tecnologia Quimica e Biologica, PORTUGAL

## Abstract

Significant advancements in the field of protein structure prediction have necessitated the need for objective and robust evaluation of protein structural models by comparing predicted models against the experimentally determined native structures to quantitate their structural similarities. Existing protein model versus native similarity metrics either consider the distances between alpha carbon (Cα) or side-chain atoms for computing the similarity. However, side-chain orientation of a protein plays a critical role in defining its conformation at the atomic-level. Despite its importance, inclusion of side-chain orientation in structural similarity evaluation has not yet been addressed. Here, we present SPECS, a side-chain-orientation-included protein model-native similarity metric for improved evaluation of protein structural models. SPECS combines side-chain orientation and global distance based measures in an integrated framework using the united-residue model of polypeptide conformation for computing model-native similarity. Experimental results demonstrate that SPECS is a reliable measure for evaluating structural similarity at the global level including and beyond the accuracy of Cα positioning. Moreover, SPECS delivers superior performance in capturing local quality aspect compared to popular global Cα positioning-based metrics ranging from models at near-experimental accuracies to models with correct overall folds—making it a robust measure suitable for both high- and moderate-resolution models. Finally, SPECS is sensitive to minute variations in side-chain χ angles even for models with perfect Cα trace, revealing the power of including side-chain orientation. Collectively, SPECS is a versatile evaluation metric covering a wide spectrum of protein modeling scenarios and simultaneously captures complementary aspects of structural similarities at multiple levels of granularities. SPECS is freely available at http://watson.cse.eng.auburn.edu/SPECS/.

## Introduction

The biological function of a protein molecule is intimately linked to its three dimensional (3D) structure. The knowledge of the 3D structure of a protein, therefore, helps us in understanding its function [1] and enables improved drug design [2]. However, experimental determination of the 3D structure of a protein is expensive and time consuming. Furthermore, the rapid accumulation of protein sequence data without available structures make it practically impossible to solve the structures of all the proteins experimentally [3]. Protein structure prediction methods aim to address these challenges by computationally predicting the 3D structure of proteins in a time-efficient manner. Computational protein 3D structure prediction, therefore, has become an integral part of structural bioinformatics [4]. Contemporary protein structure prediction methods [5–10] typically generate a large number of protein models for a given target protein and select a finite subset (typically 5 to 10) of chosen models as candidates for the final prediction. The evaluation of the accuracy of these candidate predicted models via 3D structure comparison approaches, in which the predicted models are compared against the experimentally solved native conformation of the protein in order to quantitate their similarities or differences, is critically important [11] for assessing the success of the structure prediction pipelines.

A number of model vs. native comparison-based accuracy evaluation measures have been developed over the last decade [12]. Majority of the existing model-native evaluation measures rely on superposition-based or superposition free distance-based measures [13–17], in which degrees of similarities or differences are determined based on the corresponding distances between either the main chain atoms or the side-chain atoms of the model and native. Cα Root Mean Square Deviation (Cα RMSD) [18] is one of the most commonly used main chain superposition-based model-native dissimilarity scores. It is the measure of the overall disagreement between the Cα atoms of the corresponding residues after optimal structural superposition. The lower the Cα RMSD, the better the model is in agreement with respect to the native. RMSD can be extended to include all the backbone atoms or even all atoms. However, one major limitation of RMSD is its dependence on the length of the target protein in that it is easier to obtain lower RMSD values for smaller proteins compared to larger proteins. Furthermore, RMSD is overly sensitive to minute modeling errors such as in the flexible loop regions of the structure [12].

LG-score [19] is a popular superposition-based model-native similarity metric proposed by Levitt and Gerstein. It is measured as the sum of the reciprocated distances between the aligned Cα atoms minus gap penalties. Siew, Elofsson, Rychlewski and Fischer proposed MaxSub score [20] by identifying the maximum substructure in which the distances between equivalent residues of two structures after superposition are below some threshold value, such as 3.5Å. MaxSub score lies between 0 and 1 with higher scores indicating better agreement between the model and the native. Zhang and Skolnick developed TM-score [14] by exploiting a length-dependent normalizing distance scale to eliminate the inherent protein size dependence. TM-score lies between 0 and 1, with higher scores indicating better model-native similarity.

Global Distance Test (GDT) [13], a popular structural superposition-based global model-native similarity metric, on the other hand, uses a distance threshold based approach. It is defined by the average proportion of model residues having their Cα atom distances from the corresponding residues in the native structure below a few predefined distance thresholds. Multiple superpositions of the pair of structures, each including the largest set of superimposable atoms are considered and the maximal residue set for each cutoff is selected, followed by averaging over several predetermined thresholds. For GDT-TS [13], predetermined thresholds of 1, 2, 4 and 8Å are considered for calculation of the maximal residue set. The high accuracy version of the GDT measure, GDT-HA [17], uses lower thresholds of 0.5, 1, 2 and 4Å for the calculation of the maximal residue set. The range of GDT-TS and GDT-HA measures are from 0 to 1 with higher scores indicating better agreement of the models compared to the native. GDT-TS and GDT-HA are widely used assessment metrics in the Critical Assessment of protein Structure Prediction (CASP) experiments [21,22].

LG-score, MaxSub score, TM-score, GDT-TS or GDT-HA consider only the main chain Cα atoms for quantitating the structural similarity. However, protein side-chains play a major role in defining its conformation at the atomic detail. Therefore, quantifying the side-chain similarities or differences can improve the sensitivities of model-native similarity metrics [23]. Global Distance Calculation for Side-Chains (GDC-SC) [24] is a measure, which determines the correctness of the side-chain positioning. GDC-SC metric is similar to GDT-TS in that it uses a characteristic atom for each residue type instead of relying on the Cα atom. Similar to GDT-TS, GDC-SC computes the optimal structural superposition based on the Cα atoms of the model vs. native, and subsequently uses the residue-specific side-chain characteristic atom using a distance threshold based approach to quantitate model-native similarity scores. The range of GDC-SC is from 0 to 1 with higher scores indicating better model-native similarity. Although, GDC-SC quantifies the positioning of the side-chain, it only takes into consideration the distances between the side-chain atoms and not their orientation with respect to the backbone–crucial for highly sensitive structural and functional studies based on protein structures that mandates atomistic resolution [25–27]. While existing model-native similarity measures such as LG-score, MaxSub score, TM-score, GDT-TS or GDT-HA, and GDC-SC consider either Cα atom distances or side-chain distances, an integrated structural similarity metric that can simultaneously capture the distances between the backbone and side-chain atoms as well as the orientation of side-chain atoms with respect to backbone may offer some advantages.

Here, we integrate side-chain (SC) orientation and global distance based metrics to propose a new superposition-based model-native similarity metric, Superposition-based Protein Embedded Cα-SC (SPECS) score. SPECS integrates global Cα positioning based distance and side-chain distance and orientation in a singular framework using the united-residue representation [28] for an integrated model-native similarity metric. To the best of our knowledge, this is the first study to propose a protein model evaluation metric that includes side-chain orientation. Furthermore, the seamless integration of Cα and SC in the united-residue representation is novel. Experimental results demonstrate that SPECS is a reliable and sensitive model-native similarity measure across a wide range of protein modeling scenarios in that SPECS not only is a reliable measure for evaluating the accuracy of global Cα positioning but also captures other aspects of model-native accuracy at the global level beyond just the realm of Cα positioning. Moreover, SPECS captures local quality aspect better than some of the most popular global Cα positioning-based metrics, for both high-resolution models at near-experimental accuracy and moderate-resolution models with correct backbone positioning. Finally, SPECS successfully captures minute variations of side-chain χ angles even for protein models having perfect Cα trace–revealing the effectiveness of including side-chain orientation. Collectively, SPECS is a reliable and sensitive evaluation metric for improved assessment of protein models covering a wide range of modeling scenarios and is highly effective at simultaneously capturing structural aspects at both global and local levels, thereby being a valuable new measure for comprehensive evaluation of protein structural models.

## Materials and methods

### United-residue representation for structural alignment

**Fig 1:**
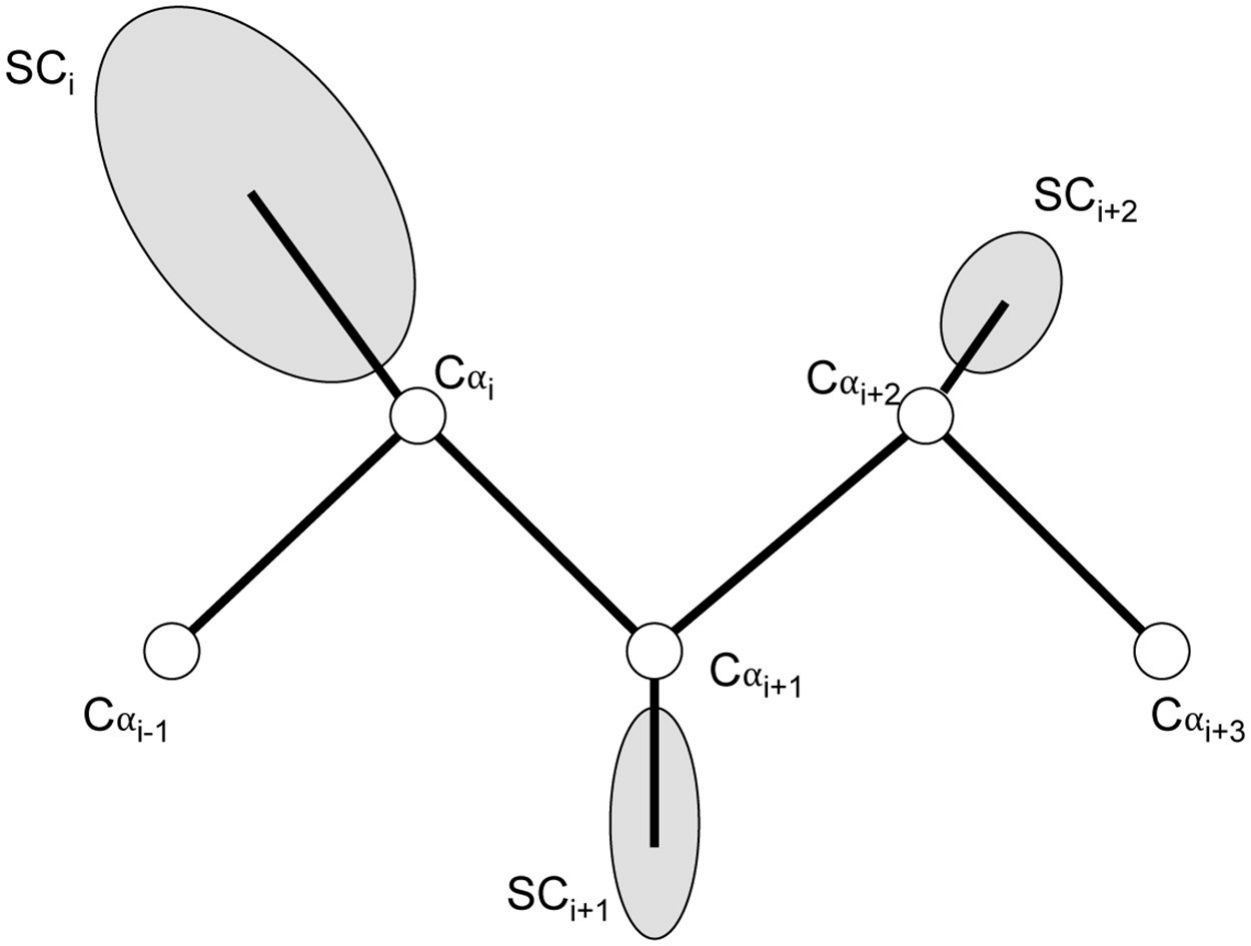
United-residue representation of polypeptide conformation. The polypeptide chain of a protein is represented as a sequence of Cα atoms and SC atoms, which are attached to the Cα atoms. All the atoms in the united-residue representation are connected using virtual bonds.

We use the united-residue representation of polypeptide conformation [28] as shown in Fig 1. In united-residue representation, the polypeptide chain of the protein is represented by a sequence of Cα atoms and characteristic side-chain (SC) atoms, which are attached to the Cα atoms. The side-chain characteristic atom is obtained by computing the centroid of all the heavy atoms present in the side-chain of a given residue in its all-atom representation. All atoms of the polypeptide chain in united-residue representation are connected using virtual bonds. The location of the residue i in the polypeptide chain is completely defined by the positioning of Cα_i_ and positioning of the corresponding side-chain characteristic atom SC_i_ attached to Cα_i_.

**Fig 2:**
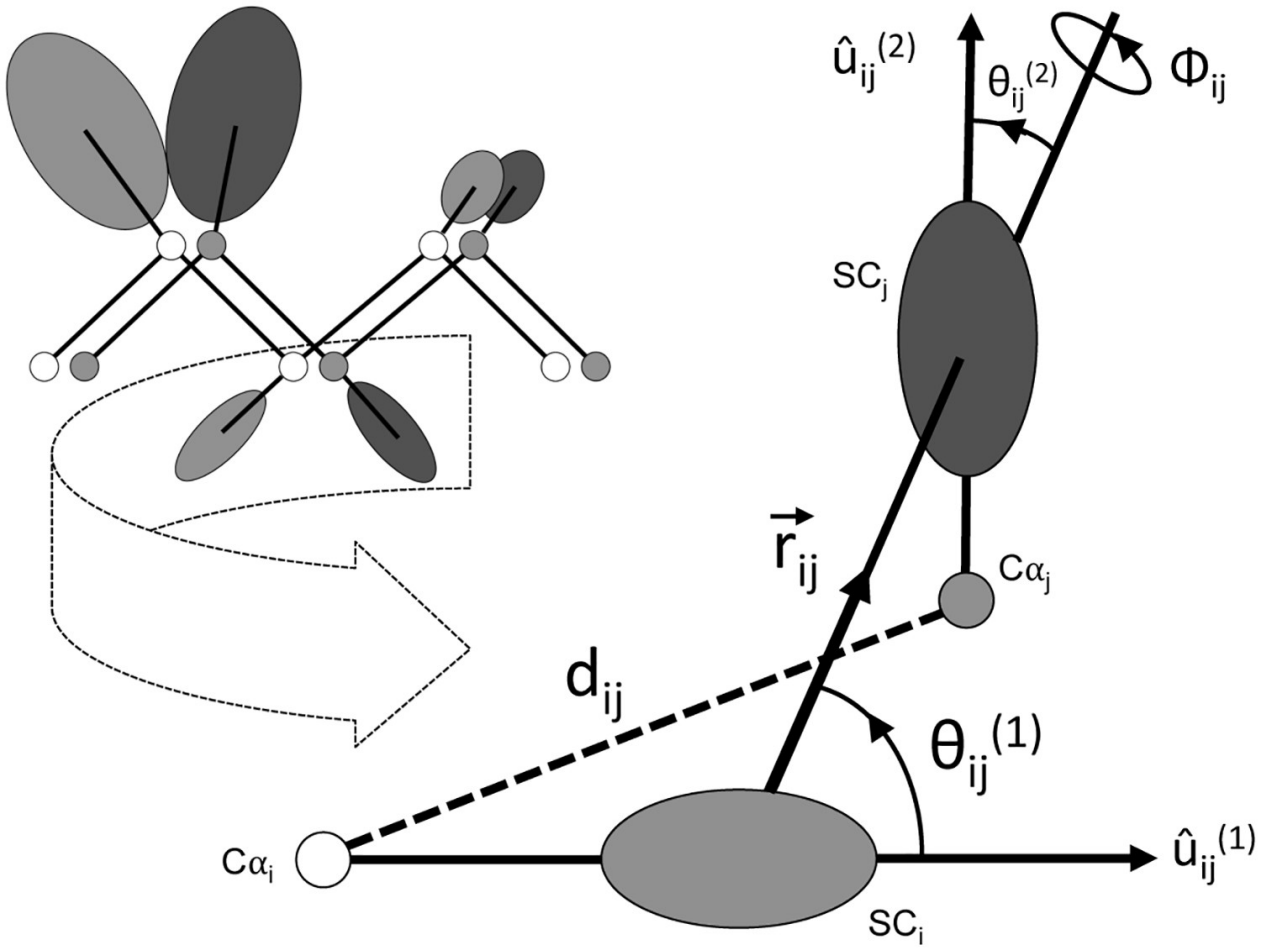
Parameterization of structurally aligned model and native structures in the united-residue representation. The structural alignment between the residue i in the model and the corresponding aligned residue j in the native is fully captured by two distances d_ij_ and r_ij_, two planar angles θ_ij_^(1)^ and θ_ij_^(2)^, and one dihedral angle Φ_ij_.

In Fig 2, we show structurally aligned model and native structures in the united residue representation. We represent the Cα position of the residue i in the model as Cα_i_ and the corresponding aligned residue j in the native as Cα_j_. Consequently, the corresponding side-chain characteristic atom i in the model is represented as SC_i_ and the characteristic atom j in the native is represented as SC_j_. While the distance between Cα_i_ and Cα_j_ is denoted purely by their Euclidean distance d_ij_, the relative positioning between the side-chain characteristic atoms is represented by the vector ${\vec{r}}_{ij}$, the magnitude of which is their Euclidean distance r_ij_. û_ij_^(1)^, û_ij_^(2)^ are the unit vectors, which represent the direction of the C_α_ and SC virtual bonds in the model and native. θ_ij_^(1)^ is the virtual planar angle between û_ij_^(1)^ and ${\vec{r}}_{ij}$ in the model and θ_ij_^(2)^ is the virtual planar angle between û_ij_^(2)^ and ${\vec{r}}_{ij}$ in the native and they are computed as follows [28]:

$$\theta_{ij}^{\left(1\right)}={\text{cos}}^{-1}\left({\hat{u}}_{ij}^{\left(1\right)}.\vec{r_{ij}}\right)$$ (1)

$$\theta_{ij}^{\left(2\right)}={\text{cos}}^{-1}\left({\hat{u}}_{ij}^{\left(2\right)}.\vec{r_{ij}}\right)$$ (2)

Φ_ij_ is the virtual dihedral angle of counterclockwise rotation between û_ij_^(2)^ and ${\vec{r}}_{ij}$ in the plane defined by û_ij_^(1)^ and ${\vec{r}}_{ij}$ and is computed as follows [28]:

$$\emptyset_{ij}={\text{cos}\;}^{-1}\left(\frac{{\hat{u}}_{ij}^{\left(1\right)}.{\hat{u}}_{ij}^{\left(2\right)}-\text{cos}\;\theta_{ij}^{\left(1\right)}\text{cos}\;\theta_{ij}^{\left(2\right)}}{\text{sin}\;\theta_{ij}^{\left(1\right)}\text{sin}\;\theta_{ij}^{\left(2\right)}}\right)$$ (3)

Structural alignment between model and native in the united residue representation, therefore, is fully captured by two distances d_ij_ and r_ij_, two planar angles θ_ij_^(1)^ and θ_ij_^(2)^, and one dihedral angle Φ_ij_.

### Formulating the side-chain-orientation-included structural similarity metric: SPECS

We utilize the aforementioned united residue representation to formulate side-chain-orientation-included structural similarity metric called SPECS, which stands for Superposition-based Protein Embedded CA SC score. SPECS is a weighted combination of five different components consisting of two distance components based on d_ij_ and r_ij_, two planar angle components based on θ_ij_^(1)^ and θ_ij_^(2)^, and one dihedral angle component based on Φ_ij_.

For computing the first component of SPECS, the optimal structural superposition between model and native is determined based on the Cα atom positioning, in order to calculate their Euclidean distances, d_ij_. Average proportion of model residues having Cα atom distances from the corresponding residues in the native structure below four different distance thresholds of 0.5, 1, 2 and 4Å are then calculated, followed by averaging the proportion of residues in four different distance thresholds as:

$$SPECS_{dCA}=\frac{p_{dCA\_05}+p_{dCA\_1}+p_{dCA\_2}+p_{dCA\_4}}{4.0}$$ (4)

where p_dCA_05_, p_dCA_1_, p_dCA_2_ and p_dCA_4_ are the proportions of the set of residues for which d_ij_ values are below distance thresholds of 0.5, 1, 2 and 4Å, respectively. Consequently SPECS_dCA_, ranges from [0, 1] with higher values indicating better model-native similarity in terms of Cα atom distances.

For computing the remaining four components of SPECS, we rely on the optimal structural superposition previously determined based on the positioning of the Cα atoms of the model vs. native to rotate and translate the side-chain atoms using the Cα positioning-based rotation and translation matrices. For the side-chain distance based component of SPECS, Euclidean distances between the aligned SC atoms in model and native, r_ij_, are calculated. Subsequently each r_ij_ value is assigned to a distance bin i, with i = 1 corresponding to values < = 0.5Å and i = 10 corresponding to values < = 5.0Å, followed by averaging the proportion of residues in ten different distance bins as:

$$SPECS_{rSC}=\frac{2\sum_{i=1}^{10}(k-i+1){p_{rsc}}_{i}}{k|k+1)}$$ (5)

where k = 10 is the number of bins and p_rSCi_ is the proportion of reference atoms assigned to distance bin i. It should be noted here that a reference atom assigned to a lower distance bin based on its r_ij_ value is, by definition, also assigned to higher distance bins. For example, if the r_ij_ value of a reference atom is less than 0.5Å, it would be assigned to all the ten bins. SPECS_rSC_ also ranges from [0, 1] with higher values indicating better model-native similarity in terms of SC atom distances.

Next, for computing the side-chain planar angle based components, we divide the θ_ij_^(1)^ and θ_ij_^(2)^ planar angles into four planar angle bins of < = 30°, < = 60°, < = 90° and < = 120°, followed by averaging the proportion of residues in four different planar angle bins as:

$$SPECS_{\theta^{\left(1\right)}}=\frac{2\sum_{i=1}^{4}(k-i+1){p_{\theta^{\left(1\right)}}}_{i}}{k(k+1)}$$ (6)

where k = 4 is the number of bins and p_θ_^(1)^_i_ is the proportion of residues assigned to planar angle bin i.

$$SPECS_{\theta^{\left(2\right)}}=\frac{2\sum_{i=1}^{4}(k-i+1){p_{\theta^{\left(2\right)}}}_{i}}{k(k+1)}$$ (7)

where k = 4 is the number of bins and p_θ_^(2)^_i_ is the proportion of residues assigned to planar angle bin i. Analogous to the distance bins, a residue belonging to a lower planar angle bin automatically falls in all higher planar angle bins. For example, if a residue’s θ_ij_^(1)^ value is less than 30°, the residue would be assigned to all the four bins in Eq 6. Also, if a residue’s θ_ij_^(2)^ value is less than 30°, the residue would be assigned to all the four bins in Eq 7.

Once again, SPECS_θ_^(1)^ and SPECS_θ_^(2)^ also range from [0, 1] with higher values indicating better model-native similarity in terms of the planar angle components of the side-chain orientations.

Next, for computing the side-chain dihedral angle based component, we divide the Φ_ij_ dihedral angle into ten bins of <= 30°, <= 60°, <= 90°, <= 120°, <= 150°, <= 180°, <= 201°, <= 240°, <= 270° and <= 300°, followed by averaging the proportion of residues in ten different dihedral angle bins as shown below:

$$SPECS_{\emptyset }=\frac{2\sum_{i=1}^{10}(k-i+1){p_{\emptyset }}_{i}}{k(k+1)}$$ (8)

where k = 10 is the number of bins and p_Φi_ is the proportion of residues assigned to dihedral angle bin i. Of note, the assignment of a residue to a lower dihedral angle bin automatically qualifies the residue to be assigned to all higher dihedral angle bins. For instance, if a residues’ Φ_ij_ value is less than 30°, the residue would be assigned to all the ten bins. Once again, SPECS_Φ_ also ranges from [0, 1] with higher values indicating better model-native similarity in terms of the dihedral angle component of the side-chain orientations.

Finally, SPECS is calculated as a weighted average of SPECS_dCA_, SPECS_rSC_, SPECS_θ_^(1)^, SPECS_θ_^(2)^, and SPECS_Φ_ as:

$$SPECS=\frac{4*SPECS_{dCA}+SPECS_{rSC}+SPECS_{\theta^{\left(1\right)}}+SPECS_{\theta^{\left(2\right)}}+SPECS_{\emptyset }\;}{8.0}$$ (9)

In this scoring scheme, equal weights are assigned to both the main chain and the side-chain based components, to equally emphasize the importance of Cα and SC positioning. The Cα distance based component, SPECS_dCA_ is given a weight of 4, which makes half of the overall score and the four side-chain based components make the other half.

### Datasets and similarity metrics used for benchmarking

We benchmark SPECS against four datasets. The first dataset is the CASP12 [29] and CASP13 regular target sets consisting of 55 and 32 regular domains for CASP12 and CASP13, respectively, with publicly available experimental structures. We use this dataset to compare SPECS against three popular model-native similarity metrics: GDT-TS [13], TM-score [14] and SphereGrinder [30]. GDT-TS and TM-score are both superposition-based global similarity scores, which determine the model-native similarity based on the distances between the Cα atoms. SphereGrinder is based on an all-atom RMSD fit between the model and native structures, using a sphere constructed by considering the set of atoms within 6Å of the Cα atoms for each residue in the native structure.

The second dataset is the CASP12 [29] and CASP13 refinement target sets consisting of a total of 37 refinement target domains with publicly available experimental structures. We use this set to compare SPECS against four high-resolution model-native similarity metrics: GDT-HA [17], CAD-AA (all atoms) [31], GDC-SC [24] and lDDT [15]. GDT-HA is a superposition-based score, which determines the model-native similarity based on the distances between the Cα atoms. GDC-SC is a superposition-based score, which determines the model-native similarity based on the distances between the side-chain characteristic atoms. CAD-AA and lDDT are all-atom based superposition-free scores.

The third dataset is the 3DRobot [32] decoy set, which consists of 200 non-homologous protein targets each having 300 decoys. 3DRobot generates a well-packed decoy pool with an even distribution of decoy accuracy over the Root Mean Square Deviation (RMSD) space with respect to the native. We use this set to evaluate the agreement between SPECS and MolProbity [33] as a local structure quality estimator and compare with two Cα atom based model-native similarity metrics GDT-HA score and TM-score. MolProbity is a log-weighted combination of the clash score, percentage of Ramachandran not favored and the percentage of bad side-chain rotamers, giving one number that reflects the crystallographic resolution at which those values would be expected. Thus, lower MolProbity scores indicate enhanced stereochemistry and better physical realism. It should be noted here, that unlike the other scores, MolProbity does not determine the local quality of a model by comparing it with the native. MolProbity score is not native-dependent and hence significantly distinct from the other scoring functions used in this work.

The fourth dataset is a monomeric proteins dataset [37], which consists of 229 protein models and 33,461 residues. These models have perfect Cα positioning with respect to the native, but possess varying side-chain conformations. We use this set to evaluate the ability of SPECS to capture the correctness of side-chain χ angles. Three widely-used side-chain prediction methods RASP [34], Rosetta-fixbb [35] and SCWRL4 [36] are used to rebuild the side-chain given the Cα trace [37]. RASP [34] is designed for rapid prediction of side-chain conformations by efficient elimination of atomic clashes and relaxation. Rosetta-fixbb [35] employs a Monte Carlo optimization approach to optimize the side-chain placement on a fixed backbone. SCWRL4 [36] utilizes a backbone-dependent rotamer library in conjunction with graph decomposition algorithms to solve the combinatorial side-chain packing problem. The prediction accuracies of these three methods are evaluated in terms of the Angular RMSDs of the χ1 side-chain torsion angles. The χ1 angle is the dihedral angle between the planes defined by the atoms N, Cα, Cβ, and Cγ. We first calculate χ1 angle for every residue using the PDB module [38] of the Biopython package [39] to compute the Angular RMSD at the target level, from the corresponding χ1 angles [40] as:

$$Angular\;RMSD=\sqrt{\frac{1}{n}\sum_{i}{\left(min(|x_{2i}-x_{1i}|,2\pi -|x_{2i}-x_{1i}|\right)}^{2}}$$ (10)

where x_1_ is the vector of χ1 angles for n residues in the target and x_2_ is the vector of corresponding χ1 angles for n residues in the native. Consequently, a lower Angular RMSD indicates better average accuracy in terms of side-chain dihedral angles. To facilitate a head-to-head comparison between SPECS and the average accuracy of side-chain dihedral angles, we subsequently normalize the Angular RMSD as:

NormalisedAngularRMSD=11+(AngularRMSDπ4)2 (11)

## Results and discussion

### SPECS is a reliable measure for evaluating the accuracy of global Cα positioning

**Fig 3:**
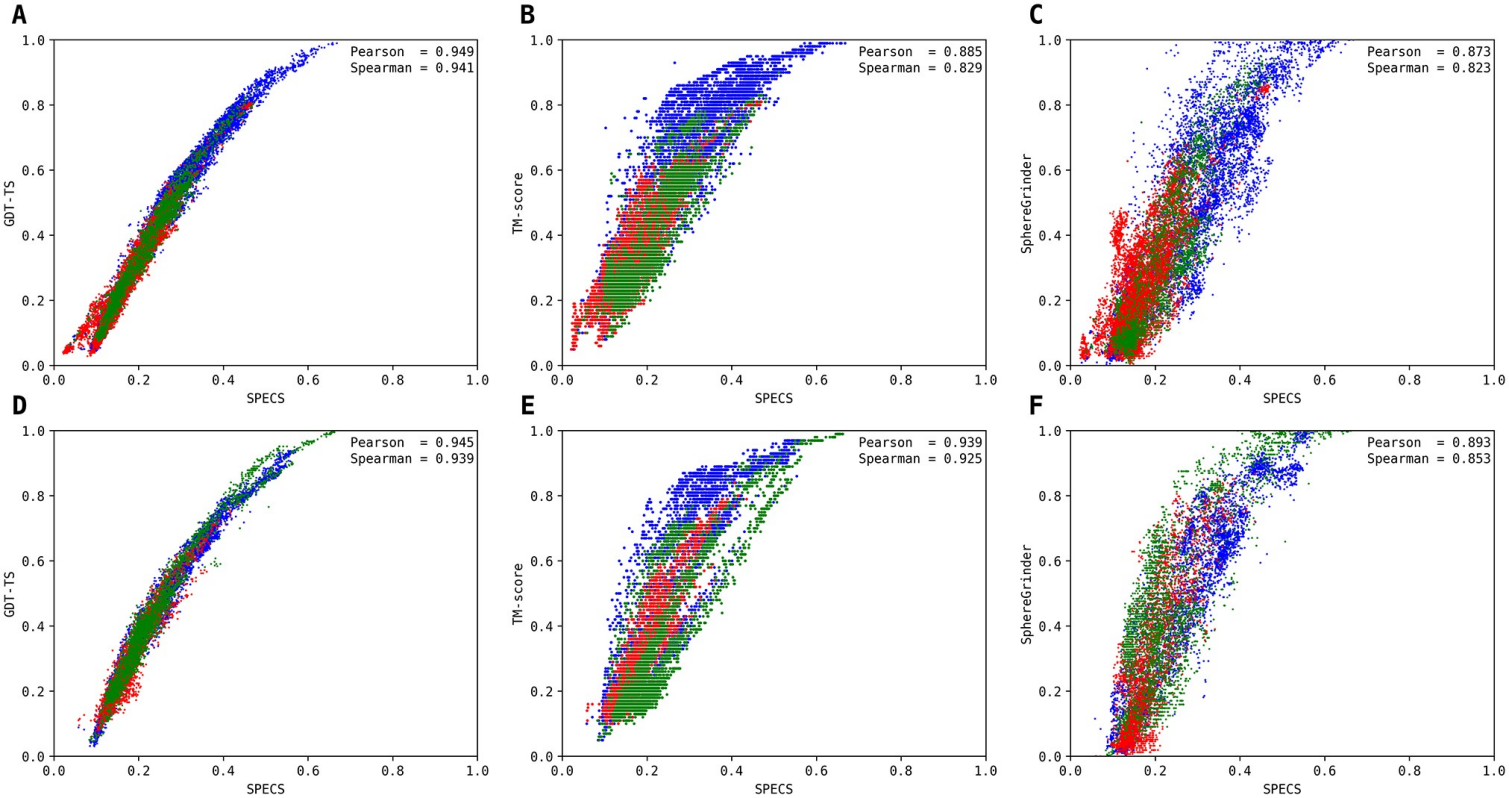
Comparisons between SPECS (horizontal axis) and GDT-TS, TM-score and SphereGrinder (vertical axis) using models in CASP12 (A-C) and CASP13 (D-F) regular single domain targets. Average Pearson (P) and Spearman (S) correlation coefficients are shown for each plot. Blue, red, and green colors represent models assessed in template-based (TBM), free modeling (FM) and overlapped (TBM/FM) categories respectively.

To investigate the ability of SPECS to quantitate model-native accuracy at the global level based on Cα positioning, we benchmark SPECS on the regular target domain from CASP12 [29] and CASP13, and compare it with the existing Cα based model-native similarity metrics. The CASP12 set consists of 55 target domains and the CASP13 decoy set consists of 32 target domains. The targets were divided into template-based (TBM), free modeling (FM) and overlapped (TBM/FM) categories as defined by the assessors. GDT-TS [13], TM-score [14] and SphereGrinder [30] are directly taken from the data archive of the Prediction Center (http://www.predictioncenter.org/), whereas SPECS is calculated by comparing the model with the native. Fig 3 shows the relationships between SPECS and GDT-TS, TM-score, SphereGrinder. The average Pearson and Spearman correlation coefficients, as shown in Fig 3, indicate that SPECS is highly correlated to other scores in that the average Pearson and Spearman correlations with respect to GDT-TS, TM-score and SphereGrinder are always greater than 0.8 in both CASP12 and CASP13 datasets, where SPECS attains the highest correlation with GDT-TS score. In CASP12 dataset, the average Pearson and Spearman correlation between SPECS and GDT-TS are 0.95 and 0.94 respectively followed by 0.89 and 0.83 respectively between SPECS and TM-score, followed by 0.87 and 0.82 respectively between SPECS and SphereGrinder. We find a similar trend in CASP13 dataset, where the average Pearson and Spearman correlations between SPECS and GDT-TS are both 0.94 respectively, followed by 0.94 and 0.93 respectively between SPECS and TM-score, followed by 0.89 and 0.85 respectively between SPECS and SphereGrinder. The persistency of strong correlations, therefore, demonstrates that SPECS is a reliable measure for evaluating model-native similarity at the global level, determined purely based on the accuracy of Cα positioning.

### Beyond Cα positioning: SPECS captures other aspects of accuracy at the global level

**Fig 4:**
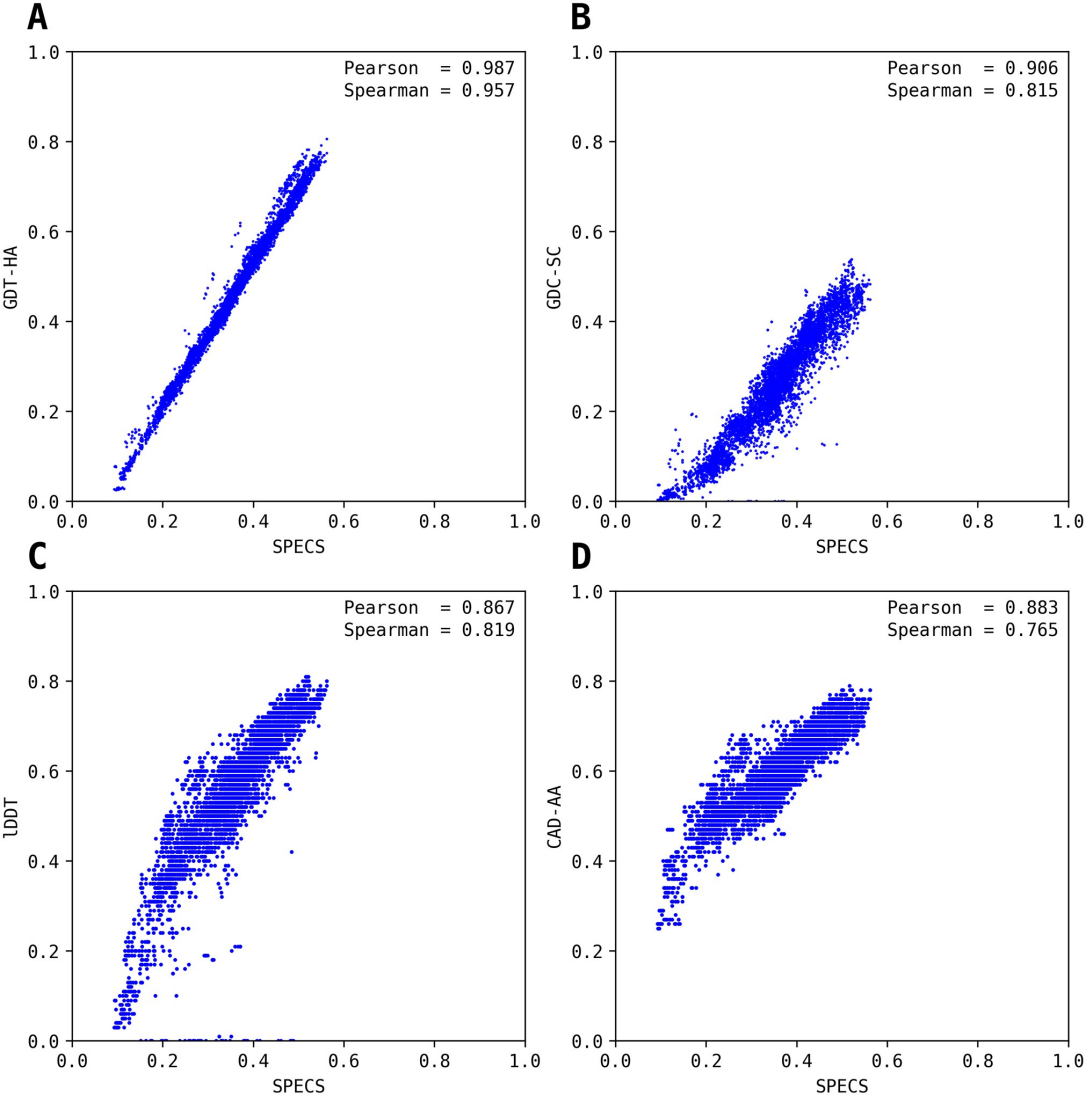
Comparisons between SPECS (horizontal axis) and existing model-native similarity metrics namely GDT-HA (A), GDC-SC (B), lDDT (C) and CAD-AA (D) (vertical axis) using models in CASP12 and CASP13 refinement targets. Average Pearson (P) and Spearman (S) correlation coefficients are shown for each plot.

To examine the ability of SPECS to capture other accuracy aspects at the global level beyond just the realm of Cα positioning, we next compare SPECS with one high-resolution Cα positioning metric GDT-HA [17], and three other metrics capturing other aspects of accuracy at the global level: (i) CAD-AA [31], based on contact area difference; (ii) GDC-SC [24] based on side-chain placement; and (iii) lDDT [15] based on local distance difference; using refinement targets from CASP12 [29] and CASP13 refinement experiments. Overall there are 37 targets (34 from CASP12 and 3 from CASP13) for which the native structures are available. Once again, GDT-HA, CAD-AA, GDC-SC and lDDT scores are taken directly from the data archive of the Prediction Center (http://www.predictioncenter.org/), whereas SPECS is calculated by comparing the model against the native. Fig 4 shows the relationships between SPECS and superposition-based scores such as GDT-HA and GDC-SC as well as superposition-free scores such as CAD-AA and lDDT. The average Pearson and Spearman correlation coefficients, as shown in Fig 4, indicate that SPECS is well-correlated to other scores in that the average Pearson and Spearman correlations always remain greater than 0.8 with the only exception between the SPECS and the lDDT having a Spearman correlation of 0.76. Similar to GDT-TS, SPECS is highly correlated with GDT-HA where the Spearman and Pearson correlations are 0.99 and 0.96 respectively. Thereafter, SPECS achieves the Pearson correlation of 0.91 with GDC-SC followed by 0.88 with CAD-AA followed by 0.87 with lDDT. Similarly, the Spearman correlation between SPECS and lDDT is 0.8 followed by 0.82 between SPECS and GDC-SC, followed by 0.76 between SPECS and CAD-AA. This strong correlation, therefore, substantiates that SPECS is not only strongly correlated with Cα positioning based accuracy metrics like GDT-HA, but also side-chain based similarity metrics like GDC-SC, and all-atom based similarity metrics like CAD-AA and lDDT. Overall, the results demonstrate the ability of the SPECS to capture other aspects of model-native accuracy at the global level including and beyond the realm of Cα positioning.

### SPECS captures local quality aspect better than global Cα based metrics

**Fig 5:**
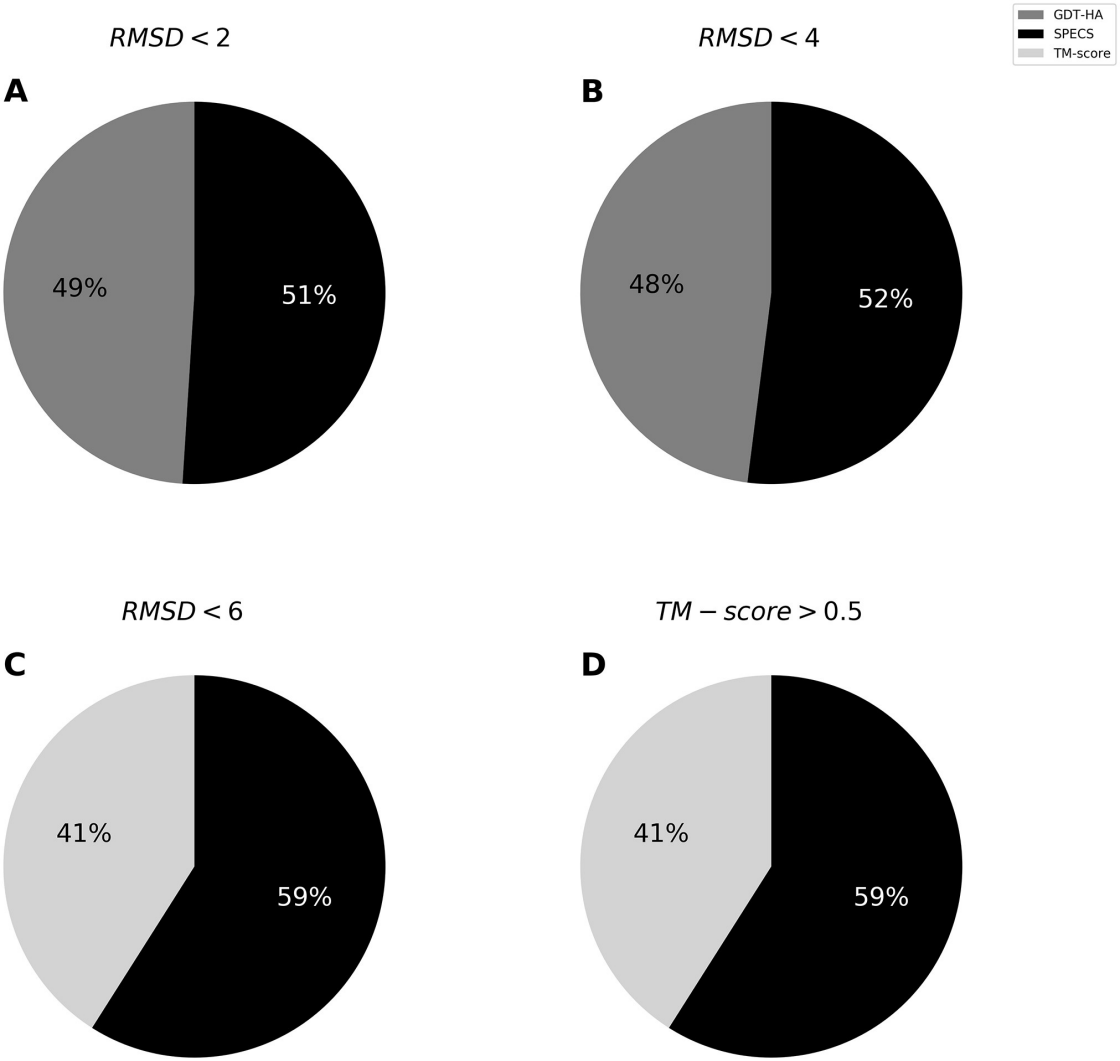
Pairs of 3DRobot models with conflicting ranking by SPECS vs. GDT-HA and TM-score with MolProbity. The 3DRobot models are divided into three bins < 2Å, < 4Å and < 6Å based on their Cα RMSD scores with respect to the natives and an additional bin consisting of decoys with TM-score > 0.5. Pie charts represent the percentages of MolProbity score agreement with rankings by SPECS vs. GDT-HA (A-B), SPECS vs. TM-score (C-D).

To assess the effectiveness of SPECS in capturing the local qualities of the models including stereochemistry and physical reasonableness, we evaluate it on 3DRobot set [32]. 3DRobot set consists of 200 non-homologous protein targets each with 300 decoys. From the entire pool consisting of 60,000 protein models, we consider models belonging to three RMSD bins namely < 2Å, < 4Å and < 6Å based on their Cα RMSD scores with respect to the natives and one TM-score bin consisting of decoys with TM-score > 0.5. The three Cα RMSD bins represent near-native accuracy, high accuracy, and medium accuracy protein models, respectively and TM-score > 0.5 represents protein models with correct overall fold. Models not belonging to any of these four bins are incorrectly folded and therefore not suitable for local quality analyses are excluded. To understand the relationship between the SPECS score assigned to a model and its physical realism, we analyze pairs of models for which SPECS vs. TM-score [14] and SPECS vs. GDT-HA [17] are in conflict. Between these conflicting pairs of models, we compare the agreement of the SPECS vs. GDT-HA and TM-score with MolProbity, which is a local quality estimator [33]. Fig 5A and 5B shows that the percentage of agreement in the ranking between SPECS and MolProbity score is consistently better compared to that between GDT-HA and MolProbity score across the < 2Å and <4Å Cα RMSD bins, indicating that SPECS is a robust measure for capturing local quality compared to GDT-HA for high-resolution protein models. Fig 5C and 5D shows that the percentage of agreement in the ranking between SPECS and MolProbity score is better compared to that between TM-score and MolProbity in < 6Å RMSD bin and when TM-score > 0.5, indicating that SPECS is a robust measure for capturing local quality compared to TM-score for moderate-resolution protein models and for those with correct overall folds. Consistently better agreement between SPECS and MolProbity in all the four bins indicates that SPECS captures local quality aspect better than global Cα positioning-based metrics, both for high- and moderate-resolution models.

### SPECS is sensitive to minute variations in side-chain χ angles

**Fig 6:**
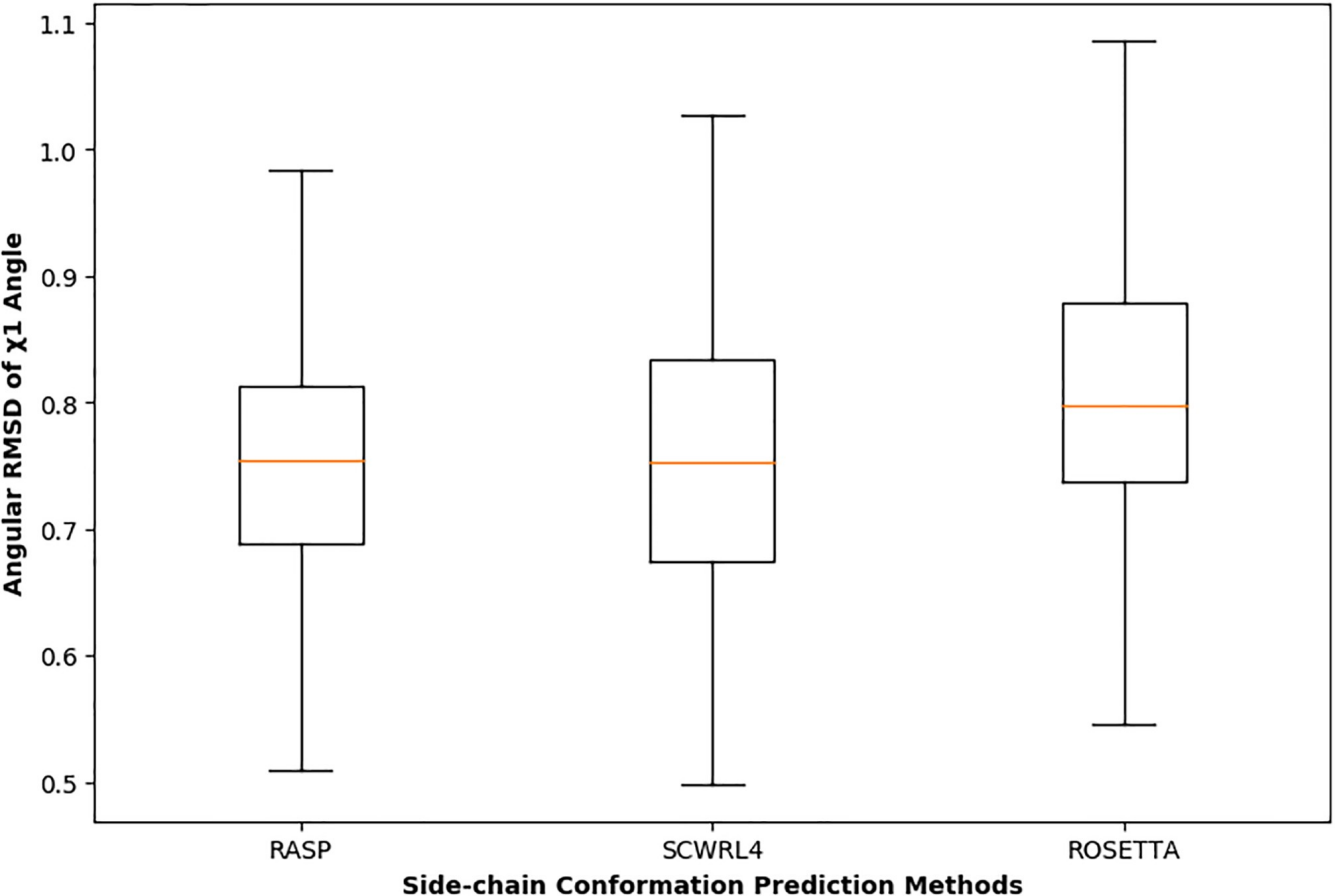
Distributions of angular RMSDs of side-chain χ angles. Lower and upper hinges: 1st and 3rd quartile. Whisker length: 1.5 times the interquartile range.

To analyze the ability of SPECS to capture variations in side-chain χ angles in models having perfect Cα trace, we analyze the side-chain χ angles of the monomeric proteins predicted by three widely used side-chain prediction methods RASP [34], Rosetta-fixbb [35], and SCWRL4 [36]. The Cα atoms of the predicted models in the dataset are perfectly aligned with respect to the native resulting in 0Å Cα-RMSDs, enabling the assessment of the structural similarity purely based on the side-chain variations. The average Angular RMSD values of the side-chain conformation predicted by the three methods are shown in Fig 6, showing the relative accuracies of the three methods based on their Angular RMSD values, with RASP ranked as the best, followed by SCWRL4, followed by Rosetta-fixbb. In Table 1, we report the correlations between SPECS and the normalized Angular RMSD values of the side-chain conformation predictions for three methods. The results demonstrate that there is a weak but positive correlation between SPECS and normalized Angular RMSD with the most accurate side-chain predictor RASP attaining the highest correlation, followed by SCWRL4, followed by Rosetta-fixbb. It should be noted here that because of the perfect Cα traces, accuracies of these models appear to be perfect (i.e., having scores of 1.0) when measured with some of the widely used structural similarity metrics such as GDT-TS, GDT-HA, and TM-score. In contrast, SPECS offers an added ability to rank these models, albeit based on the minute variations in the side-chain χ angles, thus making it more sensitive for evaluation of protein structural models.

**Table 1:** Spearman correlations between SPECS and normalized Angular RMSDs of side-chain conformation prediction methods.

Prediction Method	Spearman Correlation
RASP	0.41
SCWRL4	0.32
ROSETTA	0.30

## Conclusion

We present a side-chain-orientation included model-native similarity score, SPECS, which seamlessly combines side-chain orientation and the global distance based measures at the united-residue representation for improved assessment of protein structural models. SPECS is a weighted combination of five different components comprising of two distance based components quantifying the positioning of the Cα and SC atoms and three angle based components capturing side-chain orientation. Experimental results demonstrate that SPECS is a reliable and robust evaluation measure for protein models covering various structural aspects at both the global and local levels by being highly correlated with several global model-native similarity metrics including superposition-based scores such as GDT-TS, GDT-HA, GDC-SC, SphereGrinder, TM-score, and superposition-free scores such as CAD-AA and lDDT as well as local quality measures such as MolProbity. Moreover, SPECS offers an added ability to rank models having only minute variations in the side-chain χ angles but with perfect Cα traces, which are indistinguishable by various popular global structural similarity metrics. Collectively, these results demonstrate that SPECS is a reliable, robust, and sensitive model-native similarity metric for improved assessment of protein models that covers a wide range of protein modeling scenarios and encapsulates various aspects of structural similarity.

## Supporting information

S1 Table Target by target Pearson and Spearman correlations of SPECS with GDT-TS, TM-score and SphereGrinder scores on CASP12 regular single domain Targets. (DOCX)

S2 Table Target by target Pearson and Spearman correlations of SPECS with GDT-TS, TM-score and SphereGrinder scores on CASP13 regular single domain targets. (DOCX)

S3 Table Target by target Pearson and Spearman correlations of SPECS with GDT-HA, GDC-SC, lDDT and CAD-AA scores on CASP12 and CASP13 refinement targets. (DOCX)

S4 Table Target by target Angular RMSD of χ1 angle and SPECS on side chain conformations predicted by RASP. (DOCX)

S5 Table Target by target Angular RMSD of χ1 angle and SPECS on side chain conformations predicted by Rosetta-fixbb. (DOCX)

S6 Table Target by target Angular RMSD of χ1 angle and SPECS on side chain conformations predicted by SCWRL4. (DOCX)
