# Supplementary material for: SPECS: Integration of side-chain orientation and global distance-based measures for improved evaluation of protein structural models
Source: PLoS One. 2020 Feb 13;15(2):e0228245. doi: 10.1371/journal.pone.0228245 (PMC7018003; doi:10.1371/journal.pone.0228245)
Supplement: S1 Table — (DOCX) [file pone.0228245.s001.docx]

**Supplementary Table S1.** Target by target Pearson and Spearman correlations of SPECS with GDT-TS, TM-score and SphereGrinder scores on CASP12 regular single domain Targets.

| **Target** | **GDT-TS** | | **TM-score** | | **SphereGrinder** | |
| --- | --- | --- | --- | --- | --- | --- |
|  | **Pearson** | **Spearman** | **Pearson** | **Spearman** | **Pearson** | **Spearman** |
| T0859-D1 | 0.9116 | 0.9072 | 0.4859 | 0.4987 | 0.7389 | 0.7328 |
| T0860-D1 | 0.9971 | 0.9721 | 0.9812 | 0.8576 | 0.9889 | 0.9495 |
| T0861-D1 | 0.9845 | 0.9569 | 0.9452 | 0.9256 | 0.9613 | 0.7841 |
| T0862-D1 | 0.9651 | 0.9538 | 0.8064 | 0.7575 | 0.8658 | 0.8402 |
| T0863-D1 | 0.8522 | 0.8760 | 0.7392 | 0.6320 | 0.7921 | 0.6828 |
| T0863-D2 | 0.7498 | 0.8192 | 0.8033 | 0.6333 | 0.8391 | 0.6798 |
| T0864-D1 | 0.8681 | 0.8654 | 0.8354 | 0.7811 | 0.8350 | 0.8305 |
| T0865-D1 | 0.9629 | 0.9476 | 0.9285 | 0.9233 | 0.5801 | 0.5726 |
| T0866-D1 | 0.9874 | 0.9798 | 0.9783 | 0.9402 | 0.9783 | 0.9591 |
| T0868-D1 | 0.9702 | 0.9840 | 0.9138 | 0.9130 | 0.9176 | 0.9377 |
| T0869-D1 | 0.9263 | 0.9188 | 0.6511 | 0.6325 | 0.8303 | 0.8353 |
| T0870-D1 | 0.9231 | 0.9181 | 0.7435 | 0.7579 | 0.8286 | 0.8257 |
| T0871-D1 | 0.9923 | 0.9781 | 0.9531 | 0.9076 | 0.8772 | 0.8586 |
| T0872-D1 | 0.9896 | 0.9913 | 0.9473 | 0.9715 | 0.9655 | 0.9473 |
| T0873-D1 | 0.9941 | 0.9788 | 0.9711 | 0.8666 | 0.9752 | 0.8640 |
| T0879-D1 | 0.9876 | 0.9755 | 0.9519 | 0.8880 | 0.9619 | 0.8081 |
| T0886-D1 | 0.9175 | 0.9164 | 0.8493 | 0.7736 | 0.4908 | 0.5026 |
| T0886-D2 | 0.9805 | 0.9645 | 0.9422 | 0.8616 | 0.9542 | 0.9184 |
| T0889-D1 | 0.9926 | 0.9839 | 0.9667 | 0.9460 | 0.9555 | 0.8925 |
| T0891-D1 | 0.9909 | 0.9644 | 0.9815 | 0.9122 | 0.9724 | 0.7822 |
| T0892-D1 | 0.9855 | 0.9829 | 0.9714 | 0.9506 | 0.9394 | 0.9466 |
| T0892-D2 | 0.9475 | 0.9581 | 0.9001 | 0.9039 | 0.8882 | 0.9081 |
| T0893-D1 | 0.9529 | 0.9296 | 0.7434 | 0.6505 | 0.7378 | 0.7055 |
| T0893-D2 | 0.9850 | 0.9612 | 0.9476 | 0.9044 | 0.9521 | 0.8481 |
| T0896-D1 | 0.9864 | 0.9195 | 0.9748 | 0.8199 | 0.9135 | 0.7413 |
| T0896-D2 | 0.9873 | 0.9601 | 0.9568 | 0.8314 | 0.9561 | 0.8580 |
| T0896-D3 | 0.5576 | 0.6602 | 0.5123 | 0.4568 | 0.5085 | 0.5730 |
| T0897-D1 | 0.7386 | 0.7433 | 0.5825 | 0.4392 | 0.5860 | 0.6610 |
| T0897-D2 | 0.9738 | 0.8901 | 0.9125 | 0.6818 | 0.9046 | 0.7172 |
| T0898-D1 | 0.8942 | 0.9333 | 0.6919 | 0.7048 | 0.5711 | 0.7242 |
| T0898-D2 | 0.9683 | 0.9310 | 0.9203 | 0.8393 | 0.9243 | 0.8492 |
| T0900-D1 | 0.9699 | 0.9749 | 0.8559 | 0.8532 | 0.8593 | 0.8797 |
| T0902-D1 | 0.9875 | 0.9722 | 0.9617 | 0.9242 | 0.9510 | 0.8647 |
| T0903-D1 | 0.9907 | 0.9955 | 0.9520 | 0.9809 | 0.8306 | 0.7098 |
| T0904-D1 | 0.9763 | 0.9772 | 0.8923 | 0.8755 | 0.9134 | 0.8325 |
| T0911-D1 | 0.9870 | 0.9932 | 0.9256 | 0.8463 | 0.9328 | 0.9169 |
| T0912-D1 | 0.9901 | 0.9899 | 0.9366 | 0.9539 | 0.9738 | 0.9634 |
| T0912-D2 | 0.9860 | 0.9696 | 0.9658 | 0.9094 | 0.9424 | 0.9004 |
| T0912-D3 | 0.8984 | 0.8645 | 0.6809 | 0.4878 | 0.7130 | 0.4504 |
| T0918-D1 | 0.9641 | 0.9411 | 0.9304 | 0.9029 | 0.8935 | 0.8647 |
| T0918-D2 | 0.9506 | 0.9054 | 0.9409 | 0.8798 | 0.7804 | 0.6877 |
| T0918-D3 | 0.9773 | 0.9689 | 0.9227 | 0.8790 | 0.9407 | 0.9309 |
| T0920-D1 | 0.9916 | 0.9936 | 0.9701 | 0.9839 | 0.9532 | 0.8485 |
| T0920-D2 | 0.9931 | 0.9854 | 0.9796 | 0.9280 | 0.9846 | 0.9761 |
| T0921-D1 | 0.9944 | 0.9672 | 0.9845 | 0.8490 | 0.9847 | 0.8506 |
| T0922-D1 | 0.9879 | 0.9629 | 0.9694 | 0.9256 | 0.9651 | 0.7892 |
| T0928-D1 | 0.9888 | 0.9628 | 0.9279 | 0.8787 | 0.9276 | 0.8806 |
| T0941-D1 | 0.7139 | 0.7759 | 0.7533 | 0.5891 | 0.6452 | 0.7548 |
| T0942-D1 | 0.9940 | 0.9605 | 0.9848 | 0.9375 | 0.9841 | 0.9335 |
| T0942-D2 | 0.9858 | 0.9747 | 0.9118 | 0.8914 | 0.9273 | 0.8866 |
| T0943-D1 | 0.9783 | 0.9596 | 0.9135 | 0.8691 | 0.9446 | 0.9371 |
| T0943-D2 | 0.9896 | 0.9760 | 0.9696 | 0.9419 | 0.9519 | 0.8959 |
| T0944-D1 | 0.9908 | 0.9855 | 0.9573 | 0.9221 | 0.9834 | 0.9365 |
| T0945-D1 | 0.9888 | 0.9860 | 0.9540 | 0.9224 | 0.9763 | 0.9180 |
| T0947-D1 | 0.9903 | 0.9746 | 0.9434 | 0.9147 | 0.8720 | 0.9109 |
| **Average** | **0.9488** | **0.9407** | **0.8850** | **0.8292** | **0.8731** | **0.8228** |
