# Supplementary material for: SPECS: Integration of side-chain orientation and global distance-based measures for improved evaluation of protein structural models
Source: PLoS One. 2020 Feb 13;15(2):e0228245. doi: 10.1371/journal.pone.0228245 (PMC7018003; doi:10.1371/journal.pone.0228245)
Supplement: S2 Table — (DOCX) [file pone.0228245.s002.docx]

**Supplementary Table S2.** Target by target Pearson and Spearman correlations of SPECS with GDT-TS, TM-score and SphereGrinder scores on CASP13 regular single domain targets.

| **Target** | **GDT-TS** | | **TM-score** | | **SphereGrinder** | |
| --- | --- | --- | --- | --- | --- | --- |
|  | **Pearson** | **Spearman** | **Pearson** | **Spearman** | **Pearson** | **Spearman** |
| T0950-D1 | 0.8913 | 0.8954 | 0.8659 | 0.8520 | 0.8364 | 0.6710 |
| T0951-D1 | 0.9900 | 0.9325 | 0.9563 | 0.8775 | 0.9828 | 0.8482 |
| T0953s1-D1 | 0.9488 | 0.9443 | 0.9589 | 0.9551 | 0.8242 | 0.8471 |
| T0953s2-D1 | 0.9621 | 0.9385 | 0.9627 | 0.9558 | 0.8690 | 0.8250 |
| T0953s2-D2 | 0.9826 | 0.9745 | 0.9750 | 0.9626 | 0.9472 | 0.9371 |
| T0953s2-D3 | 0.6634 | 0.6330 | 0.6624 | 0.6300 | 0.5292 | 0.5072 |
| T0954-D1 | 0.9905 | 0.9911 | 0.9565 | 0.9749 | 0.9615 | 0.9643 |
| T0955-D1 | 0.9853 | 0.9899 | 0.9919 | 0.9923 | 0.9523 | 0.9539 |
| T0957s1-D1 | 0.9630 | 0.9568 | 0.9526 | 0.9385 | 0.8165 | 0.8618 |
| T0957s1-D2 | 0.9478 | 0.9165 | 0.9779 | 0.9407 | 0.8279 | 0.8036 |
| T0957s2-D1 | 0.9637 | 0.9773 | 0.9395 | 0.9540 | 0.9096 | 0.9447 |
| T0958-D1 | 0.9760 | 0.9832 | 0.9814 | 0.9817 | 0.9225 | 0.9275 |
| T0960-D1 | 0.7881 | 0.8102 | 0.9111 | 0.8756 | 0.7938 | 0.7144 |
| T0960-D2 | 0.9634 | 0.9602 | 0.9652 | 0.9588 | 0.9321 | 0.9029 |
| T0960-D3 | 0.9919 | 0.9879 | 0.9900 | 0.9837 | 0.9845 | 0.9695 |
| T0960-D4 | 0.8424 | 0.8643 | 0.8946 | 0.8851 | 0.8052 | 0.7746 |
| T0960-D5 | 0.9884 | 0.9790 | 0.9815 | 0.9612 | 0.9871 | 0.9445 |
| T0963-D1 | 0.7654 | 0.7746 | 0.7770 | 0.7542 | 0.5411 | 0.5005 |
| T0963-D2 | 0.9763 | 0.9585 | 0.9795 | 0.9626 | 0.9004 | 0.8715 |
| T0963-D3 | 0.9885 | 0.9886 | 0.9864 | 0.9854 | 0.9822 | 0.9709 |
| T0963-D4 | 0.8244 | 0.8095 | 0.8370 | 0.7820 | 0.8260 | 0.7642 |
| T0963-D5 | 0.9869 | 0.9864 | 0.9845 | 0.9853 | 0.9616 | 0.9584 |
| T0966-D1 | 0.9901 | 0.9830 | 0.9671 | 0.9506 | 0.9748 | 0.9376 |
| T0968s1-D1 | 0.9859 | 0.9873 | 0.9786 | 0.9812 | 0.9575 | 0.9550 |
| T0968s2-D1 | 0.9869 | 0.9849 | 0.9839 | 0.9806 | 0.9431 | 0.9531 |
| T1003-D1 | 0.9860 | 0.9800 | 0.9195 | 0.8894 | 0.9435 | 0.8233 |
| T1005-D1 | 0.9798 | 0.9827 | 0.9333 | 0.9424 | 0.9206 | 0.8805 |
| T1008-D1 | 0.9860 | 0.9667 | 0.9893 | 0.9745 | 0.9524 | 0.8675 |
| T1009-D1 | 0.9908 | 0.9911 | 0.9310 | 0.9477 | 0.9623 | 0.9278 |
| T1011-D1 | 0.9823 | 0.9780 | 0.9300 | 0.9272 | 0.9364 | 0.8538 |
| T1011-D2 | 0.9831 | 0.9885 | 0.9660 | 0.9451 | 0.9321 | 0.7925 |
| T1016-D1 | 0.9834 | 0.9577 | 0.9484 | 0.9240 | 0.9592 | 0.8328 |
| **Average** | **0.9448** | **0.9391** | **0.9386** | **0.9254** | **0.8930** | **0.8527** |
