# Supplementary material for: SPECS: Integration of side-chain orientation and global distance-based measures for improved evaluation of protein structural models
Source: PLoS One. 2020 Feb 13;15(2):e0228245. doi: 10.1371/journal.pone.0228245 (PMC7018003; doi:10.1371/journal.pone.0228245)
Supplement: S3 Table — (DOCX) [file pone.0228245.s003.docx]

**Supplementary Table S3.** Target by target Pearson and Spearman correlations of SPECS with GDT-HA, GDC-SC, lDDT and CAD-AA scores on CASP12 and CASP13 refinement targets.

| **Target** | **GDT-HA** | | **GDC-SC** | | **lDDT** | | | **CAD-AA** | |
| --- | --- | --- | --- | --- | --- | --- | --- | --- | --- |
|  | **Pearson** | **Spearman** | **Pearson** | **Spearman** | **Pearson** | **Spearman** | | **Pearson** | **Spearman** |
| TR520 | 0.9981 | 0.9860 | 0.9732 | 0.8851 | 0.9556 | 0.8465 | 0.9214 | | 0.7951 |
| TR594 | 0.9905 | 0.9648 | 0.9356 | 0.8641 | 0.8998 | 0.8208 | 0.9470 | | 0.8122 |
| TR694 | 0.9754 | 0.9660 | 0.4944 | 0.5312 | 0.9365 | 0.7633 | 0.9177 | | 0.8373 |
| TR862 | 0.9725 | 0.8497 | 0.8138 | 0.6192 | 0.7860 | 0.7113 | 0.7950 | | 0.6913 |
| TR866 | 0.9956 | 0.9647 | 0.9687 | 0.8938 | 0.9692 | 0.8947 | 0.9587 | | 0.8232 |
| TR868 | 0.9945 | 0.9375 | 0.9570 | 0.8523 | 0.9569 | 0.8289 | 0.9319 | | 0.7484 |
| TR869 | 0.9718 | 0.9279 | 0.7567 | 0.5654 | 0.7909 | 0.6344 | 0.7083 | | 0.5779 |
| TR870 | 0.9788 | 0.9417 | 0.9056 | 0.8923 | 0.6935 | 0.6840 | 0.2290 | | 0.2238 |
| TR872 | 0.9943 | 0.9303 | 0.9398 | 0.8346 | 0.9489 | 0.7888 | 0.9305 | | 0.7293 |
| TR877 | 0.9957 | 0.9374 | 0.9675 | 0.8412 | 0.9632 | 0.8953 | 0.9672 | | 0.8805 |
| TR879 | 0.9969 | 0.9811 | 0.9691 | 0.8041 | 0.9818 | 0.9126 | 0.9501 | | 0.7428 |
| TR882 | 0.9957 | 0.9735 | 0.9484 | 0.7019 | 0.9577 | 0.7563 | 0.955 | | 0.7118 |
| TR884 | 0.9815 | 0.9643 | 0.8972 | 0.8499 | 0.8584 | 0.8435 | 0.8298 | | 0.8402 |
| TR885 | 0.9971 | 0.9848 | 0.9688 | 0.8739 | 0.9415 | 0.8950 | 0.9493 | | 0.8953 |
| TR891 | 0.9981 | 0.9778 | 0.9675 | 0.8797 | 0.8789 | 0.8848 | 0.9599 | | 0.8171 |
| TR893 | 0.9977 | 0.9495 | 0.8995 | 0.8573 | 0.7647 | 0.8905 | 0.9452 | | 0.8620 |
| TR894 | 0.9862 | 0.9463 | 0.8748 | 0.7987 | 0.9344 | 0.8698 | 0.9081 | | 0.7679 |
| TR895 | 0.9965 | 0.9728 | 0.9705 | 0.8931 | 0.6713 | 0.8706 | 0.9305 | | 0.8412 |
| TR896 | 0.9975 | 0.9817 | 0.9564 | 0.8734 | 0.9388 | 0.8411 | 0.9377 | | 0.8301 |
| TR898 | 0.9450 | 0.9390 | 0.6333 | 0.6509 | 0.7365 | 0.7882 | 0.7209 | | 0.6969 |
| TR905 | 0.9916 | 0.9216 | 0.9623 | 0.7462 | 0.8686 | 0.5774 | 0.8779 | | 0.4600 |
| TR909 | 0.9963 | 0.9669 | 0.9339 | 0.6275 | 0.8651 | 0.8353 | 0.9331 | | 0.7635 |
| TR912 | 0.9964 | 0.9707 | 0.9750 | 0.9039 | 0.9252 | 0.9021 | 0.9253 | | 0.8952 |
| TR913 | 0.9967 | 0.9781 | 0.9588 | 0.8855 | 0.7692 | 0.9385 | 0.9475 | | 0.9417 |
| TR917 | 0.9965 | 0.9854 | 0.9488 | 0.8641 | 0.8866 | 0.8592 | 0.9120 | | 0.7909 |
| TR920 | 0.9971 | 0.9782 | 0.9788 | 0.9079 | 0.9730 | 0.8327 | 0.9514 | | 0.7544 |
| TR921 | 0.9968 | 0.9596 | 0.9614 | 0.8041 | 0.9031 | 0.7461 | 0.9487 | | 0.7427 |
| TR922 | 0.9934 | 0.9618 | 0.9017 | 0.8430 | 0.7934 | 0.8621 | 0.9471 | | 0.8736 |
| TR928 | 0.9972 | 0.9909 | 0.9501 | 0.8924 | 0.9248 | 0.8895 | 0.8794 | | 0.7148 |
| TR942 | 0.9901 | 0.9777 | 0.8503 | 0.6864 | 0.8335 | 0.7505 | 0.8509 | | 0.7591 |
| TR944 | 0.9981 | 0.9563 | 0.9607 | 0.8758 | 0.9723 | 0.8632 | 0.9599 | | 0.8375 |
| TR945 | 0.9971 | 0.9698 | 0.9714 | 0.8788 | 0.9478 | 0.8028 | 0.9523 | | 0.8018 |
| TR947 | 0.9964 | 0.9863 | 0.9608 | 0.886 | 0.9262 | 0.8136 | 0.8996 | | 0.8161 |
| TR948 | 0.9970 | 0.9844 | 0.9781 | 0.9242 | 0.8246 | 0.8004 | 0.8932 | | 0.7941 |
| R0968s1 | 0.9532 | 0.8925 | 0.8385 | 0.8459 | 0.7382 | 0.8451 | 0.8506 | | 0.7952 |
| R0968s2 | 0.9428 | 0.9187 | 0.8405 | 0.8408 | 0.7008 | 0.7707 | 0.7809 | | 0.7125 |
| R1016 | 0.9045 | 0.9232 | 0.7591 | 0.7921 | 0.6718 | 0.7811 | 0.7775 | | 0.7227 |
| **Average** | **0.9865** | **0.9567** | **0.9062** | **0.8153** | **0.8673** | **0.8187** | **0.8833** | | **0.7649** |
