# Supplementary material for: SPECS: Integration of side-chain orientation and global distance-based measures for improved evaluation of protein structural models
Source: PLoS One. 2020 Feb 13;15(2):e0228245. doi: 10.1371/journal.pone.0228245 (PMC7018003; doi:10.1371/journal.pone.0228245)
Supplement: S4 Table — (DOCX) [file pone.0228245.s004.docx]

**Supplementary Table S4.** Target by target Angular RMSD of χ1 angle and SPECS on side chain conformations predicted by RASP.

| **Target** | **Angular RMSD of** χ**1 Angle** | **SPECS** |
| --- | --- | --- |
| 1ah7 | 0.718252 | 0.707393 |
| 1aho | 0.666132 | 0.638157 |
| 1arb | 0.572237 | 0.716168 |
| 1atg | 0.726186 | 0.710399 |
| 1bkr | 0.77109 | 0.705524 |
| 1bx7 | 0.978036 | 0.701047 |
| 1c1k | 0.799939 | 0.699141 |
| 1c7k | 0.790452 | 0.710511 |
| 1eb6 | 0.760527 | 0.711466 |
| 1elk | 0.76867 | 0.789498 |
| 1f94 | 0.453742 | 0.663582 |
| 1g2r | 0.719479 | 0.697885 |
| 1g61 | 0.692636 | 0.711636 |
| 1g6x | 0.361655 | 0.707426 |
| 1g8a | 0.842463 | 0.704355 |
| 1gk7 | 0.937547 | 0.695659 |
| 1gmu | 0.673605 | 0.961833 |
| 1gp0 | 0.77284 | 0.707741 |
| 1i27 | 0.85861 | 0.698926 |
| 1i2t | 0.857918 | 0.699124 |
| 1i71 | 0.616527 | 0.714622 |
| 1io0 | 0.792188 | 0.704593 |
| 1jhj | 0.728141 | 0.710418 |
| 1jl1 | 0.836617 | 0.704075 |
| 1kmt | 0.75186 | 0.749934 |
| 1kng | 0.706432 | 0.707331 |
| 1koe | 0.858784 | 0.708344 |
| 1ks8 | 0.590175 | 0.713109 |
| 1lc0 | 0.837484 | 0.705243 |
| 1lmi | 0.729201 | 0.711103 |
| 1lwb | 0.765087 | 0.660125 |
| 1m4l | 0.688595 | 0.712678 |
| 1m55 | 0.773703 | 0.788577 |
| 1mf7 | 0.697897 | 0.707744 |
| 1muw | 0.774692 | 0.663498 |
| 1nc5 | 0.84565 | 0.705109 |
| 1ng6 | 0.793539 | 0.705659 |
| 1nkg | 0.725136 | 0.713401 |
| 1o06 | 0.50984 | 0.694261 |
| 1o7i | 0.63622 | 0.799496 |
| 1r6j | 0.625536 | 0.7102 |
| 1r6x | 0.836733 | 0.708166 |
| 1rju | 1.087717 | 0.701578 |
| 1roc | 0.783763 | 0.710535 |
| 1rtq | 0.681104 | 0.670107 |
| 1rtt | 0.800097 | 0.709248 |
| 1s3c | 0.690697 | 0.710005 |
| 1sau | 0.71941 | 0.703728 |
| 1t1u | 0.902159 | 0.702766 |
| 1t3y | 0.637064 | 0.707165 |
| 1t8k | 0.77951 | 0.703557 |
| 1tp6 | 0.791082 | 0.704113 |
| 1tqg | 0.814494 | 0.666742 |
| 1tua | 0.839831 | 0.701714 |
| 1ucs | 0.670254 | 0.68299 |
| 1ukf | 0.85758 | 0.705531 |
| 1vcc | 0.697725 | 0.706095 |
| 1vkk | 0.762503 | 0.702381 |
| 1w0n | 0.703989 | 0.712311 |
| 1w4s | 0.737389 | 0.708476 |
| 1wer | 0.838608 | 0.703402 |
| 1wny | 0.731243 | 0.806812 |
| 1wpa | 0.912413 | 0.696124 |
| 1x0t | 0.654473 | 0.694951 |
| 1x91 | 0.815485 | 0.703226 |
| 1xmk | 0.801679 | 0.705984 |
| 1xmt | 0.721806 | 0.709091 |
| 1xqo | 0.77948 | 0.706302 |
| 1y8a | 0.728643 | 0.70786 |
| 1yfq | 0.723826 | 0.707283 |
| 1ypy | 0.958296 | 0.781475 |
| 1yxy | 0.674668 | 0.801719 |
| 1z6n | 0.724915 | 0.710166 |
| 1zhv | 0.775462 | 0.709388 |
| 1zzk | 0.984331 | 0.702614 |
| 2c0h | 0.701545 | 0.710163 |
| 2c71 | 0.52422 | 0.715787 |
| 2ccw | 0.743689 | 0.707681 |
| 2cg7 | 0.864782 | 0.707904 |
| 2ciu | 0.923315 | 0.697385 |
| 2ckk | 0.455092 | 0.710729 |
| 2cmp | 1.008776 | 0.698661 |
| 2dsx | 0.551172 | 0.7125 |
| 2end | 0.791482 | 0.703451 |
| 2erf | 0.75511 | 0.710086 |
| 2erl | 1.101187 | 0.651562 |
| 2f23 | 0.775426 | 0.790016 |
| 2fao | 0.703966 | 0.796637 |
| 2fj8 | 0.690065 | 0.709583 |
| 2fq3 | 0.725086 | 0.708235 |
| 2g3r | 0.910735 | 0.701948 |
| 2gwm | 0.81892 | 0.706074 |
| 2h1v | 0.713935 | 0.709415 |
| 2i49 | 0.619457 | 0.712574 |
| 2i53 | 0.848109 | 0.703606 |
| 2ii2 | 0.8013 | 0.706168 |
| 2ip6 | 0.79213 | 0.70209 |
| 2ixm | 0.586053 | 0.711837 |
| 2j8b | 0.63285 | 0.707794 |
| 2jfr | 0.727171 | 0.71013 |
| 2jli | 0.834117 | 0.631114 |
| 2lis | 0.713712 | 0.701917 |
| 2mhr | 0.849503 | 0.662394 |
| 2nls | 0.681115 | 0.702178 |
| 2nuh | 0.949748 | 0.704917 |
| 2o9s | 0.738885 | 0.700611 |
| 2okt | 0.682731 | 0.709031 |
| 2ov0 | 0.623703 | 0.711948 |
| 2p51 | 0.759594 | 0.709207 |
| 2p5k | 0.876186 | 0.667154 |
| 2pnd | 0.76746 | 0.705157 |
| 2pne | 0.774294 | 0.720328 |
| 2pth | 0.727797 | 0.711004 |
| 2qcp | 0.954604 | 0.706151 |
| 2qfe | 0.809662 | 0.707987 |
| 2qjl | 0.669713 | 0.711157 |
| 2rbk | 0.550909 | 0.713628 |
| 2v9v | 0.794354 | 0.666911 |
| 2vb1 | 0.633257 | 0.662808 |
| 2vc8 | 0.697738 | 0.708333 |
| 2vq4 | 0.747877 | 0.709112 |
| 2w5q | 0.672879 | 0.709431 |
| 2wj5 | 0.829984 | 0.707741 |
| 2wmf | 0.673632 | 0.708956 |
| 2wnp | 0.696971 | 0.712207 |
| 2x3m | 0.792449 | 0.701047 |
| 2x5y | 0.894317 | 0.703529 |
| 2xbg | 0.722742 | 0.71053 |
| 2xio | 0.623125 | 0.710014 |
| 2y6h | 0.739492 | 0.711445 |
| 2y6x | 0.848447 | 0.705744 |
| 2y9u | 0.914818 | 0.703698 |
| 2yby | 0.598132 | 0.646526 |
| 2yh5 | 0.794288 | 0.707241 |
| 2z6o | 0.798523 | 0.70447 |
| 2z72 | 0.785558 | 0.708748 |
| 2znr | 0.87327 | 0.707501 |
| 3a02 | 1.109024 | 0.683422 |
| 3a07 | 0.767905 | 0.805848 |
| 3a2z | 0.768847 | 0.709007 |
| 3aj7 | 0.660774 | 0.707999 |
| 3boe | 0.561188 | 0.716382 |
| 3bwz | 0.750888 | 0.710952 |
| 3c5k | 0.745012 | 0.709628 |
| 3ca7 | 0.677215 | 0.711159 |
| 3ccd | 0.910724 | 0.794024 |
| 3chm | 0.767548 | 0.708378 |
| 3cuz | 0.677847 | 0.708404 |
| 3dfg | 0.678062 | 0.709291 |
| 3dso | 0.84892 | 0.707679 |
| 3e7r | 0.655685 | 0.702898 |
| 3e8y | 1.16872 | 0.69197 |
| 3ea6 | 0.777216 | 0.707754 |
| 3eoi | 0.585336 | 0.79478 |
| 3eye | 0.648929 | 0.711215 |
| 3f6y | 0.591765 | 0.709768 |
| 3fgh | 0.930103 | 0.700611 |
| 3fke | 0.702789 | 0.795972 |
| 3fym | 0.826562 | 0.70176 |
| 3gha | 0.714388 | 0.706684 |
| 3gkm | 0.703897 | 0.710814 |
| 3goe | 0.800455 | 0.665526 |
| 3gwi | 0.91991 | 0.703513 |
| 3h7i | 0.758941 | 0.706333 |
| 3hny | 0.670213 | 0.712078 |
| 3hpc | 0.830827 | 0.706994 |
| 3ie4 | 0.632993 | 0.822367 |
| 3ipj | 0.653622 | 0.784833 |
| 3jvl | 0.91229 | 0.696581 |
| 3k7i | 0.717311 | 0.706587 |
| 3l42 | 0.806664 | 0.693835 |
| 3lqb | 0.676885 | 0.706749 |
| 3m66 | 0.86231 | 0.701789 |
| 3mbr | 0.635113 | 0.711633 |
| 3mvs | 0.699419 | 0.707478 |
| 3ne0 | 0.720022 | 0.713456 |
| 3nir | 0.674267 | 0.66954 |
| 3onh | 0.851555 | 0.701679 |
| 3osx | 0.584627 | 0.710287 |
| 3piw | 0.754582 | 0.705261 |
| 3puc | 0.796063 | 0.66737 |
| 3qx1 | 0.83677 | 0.787823 |
| 3rjp | 0.76565 | 0.704202 |
| 3rkg | 0.802018 | 0.705867 |
| 3rt2 | 0.899518 | 0.700173 |
| 3rx9 | 0.855956 | 0.708226 |
| 3t3l | 0.725034 | 0.711739 |
| 3t7l | 0.716627 | 0.706496 |
| 3tn2 | 0.754456 | 0.705699 |
| 3tow | 0.733912 | 0.668324 |
| 3tyt | 0.745811 | 0.705414 |
| 3us6 | 0.74271 | 0.709298 |
| 3v46 | 0.792103 | 0.668629 |
| 3vmn | 0.730829 | 0.710179 |
| 3vmv | 0.615253 | 0.714762 |
| 3vor | 0.784757 | 0.666896 |
| 3zbd | 0.723619 | 0.783605 |
| 3zsu | 0.674352 | 0.708157 |
| 3zzo | 0.782864 | 0.706769 |
| 3zzp | 0.592896 | 0.707095 |
| 4a02 | 0.768409 | 0.708961 |
| 4a4j | 0.859899 | 0.706324 |
| 4a9v | 0.656743 | 0.64408 |
| 4abl | 0.744529 | 0.708771 |
| 4acj | 0.720654 | 0.641004 |
| 4ann | 0.808519 | 0.706082 |
| 4b89 | 0.692568 | 0.706675 |
| 4b9g | 0.784663 | 0.743065 |
| 4d8b | 0.643157 | 0.662983 |
| 4dvc | 0.757477 | 0.668577 |
| 4e40 | 0.75111 | 0.707987 |
| 4eb0 | 0.663383 | 0.71335 |
| 4esm | 0.660894 | 0.709824 |
| 4f1v | 0.606084 | 0.674383 |
| 4f2f | 0.676815 | 0.70642 |
| 4ftf | 0.895734 | 0.702294 |
| 4g3o | 0.768151 | 0.701372 |
| 4ga2 | 0.824324 | 0.667803 |
| 4gc3 | 0.77245 | 0.705016 |
| 4gco | 0.680965 | 0.704877 |
| 4gei | 0.878732 | 0.703164 |
| 4gmq | 0.724955 | 0.709894 |
| 4gzc | 0.718053 | 0.703487 |
| 4h4n | 0.837696 | 0.704454 |
| 4he6 | 0.755491 | 0.701456 |
| 4hu2 | 0.754857 | 0.711621 |
| 4i6x | 0.784824 | 0.705565 |
| 4iej | 0.916538 | 0.69903 |
| 4il7 | 0.774787 | 0.711765 |
