# Supplementary material for: SPECS: Integration of side-chain orientation and global distance-based measures for improved evaluation of protein structural models
Source: PLoS One. 2020 Feb 13;15(2):e0228245. doi: 10.1371/journal.pone.0228245 (PMC7018003; doi:10.1371/journal.pone.0228245)
Supplement: S5 Table — (DOCX) [file pone.0228245.s005.docx]

**Supplementary Table S5.** Target by target Angular RMSD of χ1 angle and SPECS on side chain conformations predicted by Rosetta-fixbb.

| **Target** | **Angular RMSD of** χ**1 Angle** | **SPECS** |
| --- | --- | --- |
| 1ah7 | 0.822526 | 0.733015 |
| 1aho | 0.942609 | 0.701296 |
| 1arb | 0.735152 | 0.739954 |
| 1atg | 0.7576 | 0.727278 |
| 1bkr | 0.615115 | 0.745812 |
| 1bx7 | 1.177058 | 0.728387 |
| 1c1k | 0.830994 | 0.731891 |
| 1c7k | 0.591651 | 0.739024 |
| 1eb6 | 0.924912 | 0.735882 |
| 1elk | 0.86746 | 0.811297 |
| 1f94 | 1.137395 | 0.715061 |
| 1g2r | 0.705869 | 0.726426 |
| 1g61 | 0.634967 | 0.738879 |
| 1g6x | 0.588666 | 0.745435 |
| 1g8a | 0.839501 | 0.731538 |
| 1gk7 | 1.139485 | 0.719289 |
| 1gmu | 0.694859 | 0.971558 |
| 1gp0 | 0.721717 | 0.735159 |
| 1i27 | 0.949288 | 0.723646 |
| 1i2t | 0.901598 | 0.731352 |
| 1i71 | 0.74043 | 0.741772 |
| 1io0 | 0.753105 | 0.732263 |
| 1jhj | 0.712529 | 0.745624 |
| 1jl1 | 0.845557 | 0.728013 |
| 1kmt | 0.762926 | 0.785458 |
| 1kng | 0.72421 | 0.728125 |
| 1koe | 0.751175 | 0.736747 |
| 1ks8 | 0.731113 | 0.740967 |
| 1lc0 | 0.977175 | 0.728292 |
| 1lmi | 0.781695 | 0.732616 |
| 1lwb | 0.771926 | 0.709156 |
| 1m4l | 0.7761 | 0.743504 |
| 1m55 | 0.848615 | 0.815132 |
| 1mf7 | 0.830356 | 0.738431 |
| 1muw | 0.823478 | 0.708484 |
| 1nc5 | 0.823285 | 0.737106 |
| 1ng6 | 0.741223 | 0.739765 |
| 1nkg | 0.773407 | 0.740451 |
| 1o06 | 0.789357 | 0.733523 |
| 1o7i | 0.896115 | 0.813804 |
| 1r6j | 0.653174 | 0.731693 |
| 1r6x | 0.834326 | 0.736178 |
| 1rju | 1.007044 | 0.742487 |
| 1roc | 0.932169 | 0.742016 |
| 1rtq | 0.813997 | 0.712121 |
| 1rtt | 0.72942 | 0.738885 |
| 1s3c | 0.657041 | 0.738282 |
| 1sau | 0.818401 | 0.729944 |
| 1t1u | 0.878635 | 0.731468 |
| 1t3y | 0.546178 | 0.740831 |
| 1t8k | 0.754997 | 0.738164 |
| 1tp6 | 0.842924 | 0.74467 |
| 1tqg | 0.903589 | 0.706764 |
| 1tua | 0.823392 | 0.727128 |
| 1ucs | 0.719854 | 0.710494 |
| 1ukf | 0.760882 | 0.73663 |
| 1vcc | 0.83742 | 0.73322 |
| 1vkk | 0.697778 | 0.73857 |
| 1w0n | 0.720512 | 0.738333 |
| 1w4s | 0.73377 | 0.740629 |
| 1wer | 0.87281 | 0.729795 |
| 1wny | 0.75397 | 0.819545 |
| 1wpa | 0.930686 | 0.740166 |
| 1x0t | 0.890514 | 0.716199 |
| 1x91 | 0.854256 | 0.72934 |
| 1xmk | 0.906781 | 0.72651 |
| 1xmt | 0.869879 | 0.739892 |
| 1xqo | 0.813512 | 0.732025 |
| 1y8a | 0.788844 | 0.734861 |
| 1yfq | 0.71291 | 0.739414 |
| 1ypy | 0.977566 | 0.797147 |
| 1yxy | 0.758809 | 0.814081 |
| 1z6n | 0.716469 | 0.737931 |
| 1zhv | 0.793585 | 0.730393 |
| 1zzk | 1.006304 | 0.720213 |
| 2c0h | 0.648412 | 0.743855 |
| 2c71 | 0.658258 | 0.733348 |
| 2ccw | 0.788801 | 0.735139 |
| 2cg7 | 1.14596 | 0.732879 |
| 2ciu | 0.90569 | 0.733638 |
| 2ckk | 0.646493 | 0.729905 |
| 2cmp | 0.883003 | 0.733015 |
| 2dsx | 0.842536 | 0.739707 |
| 2end | 0.691689 | 0.738296 |
| 2erf | 0.864285 | 0.73514 |
| 2erl | 0.994883 | 0.701648 |
| 2f23 | 0.802461 | 0.817178 |
| 2fao | 0.867773 | 0.811753 |
| 2fj8 | 0.910484 | 0.742888 |
| 2fq3 | 0.83031 | 0.726979 |
| 2g3r | 0.903241 | 0.731895 |
| 2gwm | 0.815572 | 0.73167 |
| 2h1v | 0.77418 | 0.739096 |
| 2i49 | 0.758178 | 0.735027 |
| 2i53 | 0.787077 | 0.732655 |
| 2ii2 | 0.772682 | 0.743518 |
| 2ip6 | 0.798483 | 0.729911 |
| 2ixm | 0.743023 | 0.737807 |
| 2j8b | 0.861346 | 0.74697 |
| 2jfr | 0.72637 | 0.73985 |
| 2jli | 0.635722 | 0.668318 |
| 2lis | 0.694011 | 0.728557 |
| 2mhr | 0.926857 | 0.704449 |
| 2nls | 0.72488 | 0.743624 |
| 2nuh | 0.980982 | 0.728813 |
| 2o9s | 0.830054 | 0.739009 |
| 2okt | 0.791335 | 0.739218 |
| 2ov0 | 0.393419 | 0.74513 |
| 2p51 | 0.800265 | 0.732509 |
| 2p5k | 0.890485 | 0.703968 |
| 2pnd | 0.864991 | 0.733585 |
| 2pne | 1.105147 | 0.712837 |
| 2pth | 0.624139 | 0.733744 |
| 2qcp | 0.933933 | 0.732358 |
| 2qfe | 0.810395 | 0.729545 |
| 2qjl | 0.710024 | 0.743629 |
| 2rbk | 0.787006 | 0.737722 |
| 2v9v | 0.823198 | 0.7058 |
| 2vb1 | 0.783939 | 0.708712 |
| 2vc8 | 0.673913 | 0.746275 |
| 2vq4 | 0.781607 | 0.748156 |
| 2w5q | 0.670523 | 0.744739 |
| 2wj5 | 0.727321 | 0.739844 |
| 2wmf | 0.856057 | 0.734369 |
| 2wnp | 0.723734 | 0.746413 |
| 2x3m | 0.931006 | 0.728532 |
| 2x5y | 0.84211 | 0.74154 |
| 2xbg | 0.679427 | 0.738304 |
| 2xio | 0.665798 | 0.732272 |
| 2y6h | 0.769297 | 0.733655 |
| 2y6x | 0.998509 | 0.732767 |
| 2y9u | 0.940361 | 0.737805 |
| 2yby | 0.887827 | 0.696665 |
| 2yh5 | 0.770817 | 0.733631 |
| 2z6o | 0.778306 | 0.730004 |
| 2z72 | 0.80604 | 0.740267 |
| 2znr | 0.917645 | 0.736977 |
| 3a02 | 0.997288 | 0.720388 |
| 3a07 | 0.992635 | 0.819681 |
| 3a2z | 0.715772 | 0.744157 |
| 3aj7 | 0.806659 | 0.738629 |
| 3boe | 0.59072 | 0.734406 |
| 3bwz | 0.792465 | 0.742424 |
| 3c5k | 0.673935 | 0.741225 |
| 3ca7 | 0.978707 | 0.743682 |
| 3ccd | 0.791091 | 0.816898 |
| 3chm | 0.79388 | 0.727894 |
| 3cuz | 0.796694 | 0.736224 |
| 3dfg | 0.762141 | 0.731834 |
| 3dso | 0.859127 | 0.736364 |
| 3e7r | 1.116193 | 0.7175 |
| 3e8y | 1.086265 | 0.730265 |
| 3ea6 | 0.821825 | 0.73824 |
| 3eoi | 0.664617 | 0.819826 |
| 3eye | 0.590798 | 0.74847 |
| 3f6y | 0.773019 | 0.736291 |
| 3fgh | 0.905628 | 0.741113 |
| 3fke | 0.791415 | 0.804139 |
| 3fym | 0.90564 | 0.733329 |
| 3gha | 0.744197 | 0.739088 |
| 3gkm | 0.796458 | 0.730334 |
| 3goe | 0.876943 | 0.704304 |
| 3gwi | 0.879013 | 0.734112 |
| 3h7i | 0.815988 | 0.737124 |
| 3hny | 0.792115 | 0.743211 |
| 3hpc | 0.806586 | 0.747045 |
| 3ie4 | 0.707679 | 0.841922 |
| 3ipj | 0.646952 | 0.805921 |
| 3jvl | 0.953925 | 0.723952 |
| 3k7i | 0.75396 | 0.74177 |
| 3l42 | 0.871855 | 0.716079 |
| 3lqb | 0.803358 | 0.730538 |
| 3m66 | 0.893717 | 0.730055 |
| 3mbr | 0.800065 | 0.734717 |
| 3mvs | 0.740137 | 0.733398 |
| 3ne0 | 0.591783 | 0.723574 |
| 3nir | 0.753231 | 0.713785 |
| 3onh | 0.761607 | 0.732965 |
| 3osx | 0.737839 | 0.732978 |
| 3piw | 0.830942 | 0.730239 |
| 3puc | 0.779376 | 0.707827 |
| 3qx1 | 0.84658 | 0.816386 |
| 3rjp | 0.916713 | 0.736162 |
| 3rkg | 0.881867 | 0.737826 |
| 3rt2 | 0.853141 | 0.734981 |
| 3rx9 | 0.91956 | 0.733191 |
| 3t3l | 0.831337 | 0.732391 |
| 3t7l | 0.733191 | 0.733768 |
| 3tn2 | 0.913104 | 0.729011 |
| 3tow | 0.843461 | 0.709368 |
| 3tyt | 0.861412 | 0.738135 |
| 3us6 | 0.792395 | 0.742499 |
| 3v46 | 0.920743 | 0.701861 |
| 3vmn | 0.756805 | 0.740394 |
| 3vmv | 0.742567 | 0.741811 |
| 3vor | 0.797622 | 0.715522 |
| 3zbd | 0.645289 | 0.810661 |
| 3zsu | 0.649721 | 0.735911 |
| 3zzo | 1.153024 | 0.731855 |
| 3zzp | 0.714469 | 0.731143 |
| 4a02 | 0.778525 | 0.731613 |
| 4a4j | 0.930719 | 0.736018 |
| 4a9v | 0.711431 | 0.711553 |
| 4abl | 0.691705 | 0.738567 |
| 4acj | 0.755506 | 0.709261 |
| 4ann | 0.925753 | 0.736519 |
| 4b89 | 0.80665 | 0.726556 |
| 4b9g | 0.783159 | 0.791719 |
| 4d8b | 0.679085 | 0.712482 |
| 4dvc | 0.738294 | 0.709604 |
| 4e40 | 0.83339 | 0.730496 |
| 4eb0 | 0.664506 | 0.736866 |
| 4esm | 0.784346 | 0.734634 |
| 4f1v | 0.629356 | 0.718209 |
| 4f2f | 0.907989 | 0.727341 |
| 4ftf | 0.947352 | 0.727304 |
| 4g3o | 0.89685 | 0.728924 |
| 4ga2 | 0.765088 | 0.708807 |
| 4gc3 | 0.792572 | 0.738295 |
| 4gco | 0.765762 | 0.732566 |
| 4gei | 0.882308 | 0.72938 |
| 4gmq | 0.583835 | 0.73482 |
| 4gzc | 0.804163 | 0.73948 |
| 4h4n | 0.880682 | 0.730004 |
| 4he6 | 0.829544 | 0.738496 |
| 4hu2 | 0.779128 | 0.733288 |
| 4i6x | 0.879504 | 0.732615 |
| 4iej | 0.801435 | 0.735742 |
| 4il7 | 0.867319 | 0.738944 |
