# Supplementary material for: SPECS: Integration of side-chain orientation and global distance-based measures for improved evaluation of protein structural models
Source: PLoS One. 2020 Feb 13;15(2):e0228245. doi: 10.1371/journal.pone.0228245 (PMC7018003; doi:10.1371/journal.pone.0228245)
Supplement: S6 Table — (DOCX) [file pone.0228245.s006.docx]

**Supplementary Table S6.** Target by target Angular RMSD of χ1 angle and SPECS on side chain conformations predicted by SCWRL4.

| **Target** | **Angular RMSD of** χ**1 Angle** | **SPECS** |
| --- | --- | --- |
| 1ah7 | 0.716829 | 0.716011 |
| 1aho | 0.765145 | 0.654616 |
| 1arb | 0.630064 | 0.721587 |
| 1atg | 0.680158 | 0.717183 |
| 1bkr | 0.63754 | 0.719139 |
| 1bx7 | 0.977612 | 0.700312 |
| 1c1k | 0.806048 | 0.712662 |
| 1c7k | 0.881045 | 0.712646 |
| 1eb6 | 0.727413 | 0.718702 |
| 1elk | 0.841992 | 0.798411 |
| 1f94 | 0.788361 | 0.669499 |
| 1g2r | 0.704945 | 0.711666 |
| 1g61 | 0.722804 | 0.727101 |
| 1g6x | 0.643742 | 0.703272 |
| 1g8a | 0.783087 | 0.723779 |
| 1gk7 | 1.147157 | 0.695484 |
| 1gmu | 0.650902 | 0.959643 |
| 1gp0 | 0.713802 | 0.721839 |
| 1i27 | 0.939642 | 0.710041 |
| 1i2t | 0.799453 | 0.70978 |
| 1i71 | 0.58701 | 0.722385 |
| 1io0 | 0.79186 | 0.719284 |
| 1jhj | 0.72053 | 0.722706 |
| 1jl1 | 0.698631 | 0.716201 |
| 1kmt | 0.85357 | 0.757428 |
| 1kng | 0.58098 | 0.719894 |
| 1koe | 0.733268 | 0.719021 |
| 1ks8 | 0.599794 | 0.720266 |
| 1lc0 | 0.904576 | 0.717347 |
| 1lmi | 0.615274 | 0.730864 |
| 1lwb | 0.635194 | 0.674218 |
| 1m4l | 0.655857 | 0.724493 |
| 1m55 | 0.667692 | 0.805982 |
| 1mf7 | 0.705376 | 0.721105 |
| 1muw | 0.710341 | 0.671885 |
| 1nc5 | 0.660369 | 0.725279 |
| 1ng6 | 0.934352 | 0.710765 |
| 1nkg | 0.685393 | 0.727541 |
| 1o06 | 0.669905 | 0.715795 |
| 1o7i | 0.77305 | 0.807431 |
| 1r6j | 0.669014 | 0.716325 |
| 1r6x | 0.773019 | 0.722324 |
| 1rju | 1.35098 | 0.697948 |
| 1roc | 0.779084 | 0.729348 |
| 1rtq | 0.676364 | 0.677976 |
| 1rtt | 0.797019 | 0.717137 |
| 1s3c | 0.653727 | 0.725008 |
| 1sau | 0.797189 | 0.711613 |
| 1t1u | 0.927449 | 0.71608 |
| 1t3y | 0.563881 | 0.723326 |
| 1t8k | 0.926444 | 0.708456 |
| 1tp6 | 0.880588 | 0.719165 |
| 1tqg | 0.911082 | 0.671364 |
| 1tua | 0.865721 | 0.717797 |
| 1ucs | 0.605339 | 0.682901 |
| 1ukf | 0.872366 | 0.718599 |
| 1vcc | 0.871093 | 0.714846 |
| 1vkk | 0.74086 | 0.718397 |
| 1w0n | 0.659816 | 0.723068 |
| 1w4s | 0.703859 | 0.722595 |
| 1wer | 0.872865 | 0.72131 |
| 1wny | 0.808053 | 0.811143 |
| 1wpa | 0.950463 | 0.695008 |
| 1x0t | 0.795297 | 0.699378 |
| 1x91 | 0.896352 | 0.715871 |
| 1xmk | 0.880931 | 0.715607 |
| 1xmt | 0.724113 | 0.714928 |
| 1xqo | 0.778104 | 0.713219 |
| 1y8a | 0.741641 | 0.722872 |
| 1yfq | 0.708574 | 0.725857 |
| 1ypy | 0.895164 | 0.78406 |
| 1yxy | 0.62241 | 0.7983 |
| 1z6n | 0.651647 | 0.723912 |
| 1zhv | 0.795453 | 0.73041 |
| 1zzk | 0.998854 | 0.701861 |
| 2c0h | 0.714885 | 0.724511 |
| 2c71 | 0.587175 | 0.726458 |
| 2ccw | 0.732068 | 0.721591 |
| 2cg7 | 0.895243 | 0.709861 |
| 2ciu | 0.93227 | 0.716842 |
| 2ckk | 0.608355 | 0.720975 |
| 2cmp | 1.106961 | 0.696287 |
| 2dsx | 1.026983 | 0.716324 |
| 2end | 0.803688 | 0.714283 |
| 2erf | 0.763215 | 0.720514 |
| 2erl | 0.967576 | 0.660767 |
| 2f23 | 0.771448 | 0.799063 |
| 2fao | 0.680102 | 0.795855 |
| 2fj8 | 0.708472 | 0.718333 |
| 2fq3 | 0.683559 | 0.71492 |
| 2g3r | 0.860733 | 0.717714 |
| 2gwm | 0.804502 | 0.717057 |
| 2h1v | 0.817036 | 0.719969 |
| 2i49 | 0.53626 | 0.725148 |
| 2i53 | 0.783132 | 0.715274 |
| 2ii2 | 0.782018 | 0.718178 |
| 2ip6 | 0.753272 | 0.711638 |
| 2ixm | 0.73404 | 0.725375 |
| 2j8b | 0.553083 | 0.719945 |
| 2jfr | 0.640109 | 0.728399 |
| 2jli | 0.862135 | 0.662375 |
| 2lis | 0.65896 | 0.709074 |
| 2mhr | 0.825212 | 0.675655 |
| 2nls | 0.610211 | 0.705429 |
| 2nuh | 0.9463 | 0.726617 |
| 2o9s | 0.614057 | 0.720132 |
| 2okt | 0.692249 | 0.724578 |
| 2ov0 | 0.582656 | 0.72039 |
| 2p51 | 0.768137 | 0.72246 |
| 2p5k | 0.891805 | 0.666685 |
| 2pnd | 0.800179 | 0.72034 |
| 2pne | 0.98443 | 0.706019 |
| 2pth | 0.687291 | 0.723269 |
| 2qcp | 0.895337 | 0.719744 |
| 2qfe | 0.864619 | 0.720389 |
| 2qjl | 0.772633 | 0.727904 |
| 2rbk | 0.617092 | 0.720346 |
| 2v9v | 0.760713 | 0.668333 |
| 2vb1 | 0.552137 | 0.671327 |
| 2vc8 | 0.684927 | 0.726862 |
| 2vq4 | 0.700745 | 0.722749 |
| 2w5q | 0.640566 | 0.720044 |
| 2wj5 | 0.794372 | 0.728622 |
| 2wmf | 0.660305 | 0.724988 |
| 2wnp | 0.646286 | 0.732305 |
| 2x3m | 0.874255 | 0.716546 |
| 2x5y | 0.865558 | 0.714759 |
| 2xbg | 0.679973 | 0.727048 |
| 2xio | 0.675039 | 0.723231 |
| 2y6h | 0.645703 | 0.732927 |
| 2y6x | 0.924941 | 0.714804 |
| 2y9u | 0.984947 | 0.720047 |
| 2yby | 0.698429 | 0.665826 |
| 2yh5 | 0.77997 | 0.71775 |
| 2z6o | 0.76331 | 0.718339 |
| 2z72 | 0.81127 | 0.723487 |
| 2znr | 0.823539 | 0.719452 |
| 3a02 | 1.023194 | 0.700245 |
| 3a07 | 0.639101 | 0.81009 |
| 3a2z | 0.670145 | 0.731752 |
| 3aj7 | 0.653504 | 0.721721 |
| 3boe | 0.616475 | 0.719068 |
| 3bwz | 0.857066 | 0.719218 |
| 3c5k | 0.720398 | 0.729419 |
| 3ca7 | 0.878582 | 0.714068 |
| 3ccd | 0.898655 | 0.807139 |
| 3chm | 0.815262 | 0.721125 |
| 3cuz | 0.715724 | 0.720401 |
| 3dfg | 0.762841 | 0.709611 |
| 3dso | 0.968658 | 0.710675 |
| 3e7r | 0.719548 | 0.710994 |
| 3e8y | 1.101717 | 0.694924 |
| 3ea6 | 0.76357 | 0.717955 |
| 3eoi | 0.568864 | 0.802227 |
| 3eye | 0.63275 | 0.729739 |
| 3f6y | 0.651703 | 0.726509 |
| 3fgh | 1.074775 | 0.687059 |
| 3fke | 0.742218 | 0.786641 |
| 3fym | 0.733592 | 0.714717 |
| 3gha | 0.819338 | 0.717418 |
| 3gkm | 0.6849 | 0.722923 |
| 3goe | 0.777055 | 0.67696 |
| 3gwi | 0.767642 | 0.723912 |
| 3h7i | 0.796696 | 0.718283 |
| 3hny | 0.737133 | 0.734499 |
| 3hpc | 0.793096 | 0.7275 |
| 3ie4 | 0.607465 | 0.830952 |
| 3ipj | 0.657553 | 0.787189 |
| 3jvl | 0.86831 | 0.70721 |
| 3k7i | 0.617828 | 0.723118 |
| 3l42 | 0.67011 | 0.711865 |
| 3lqb | 0.767951 | 0.715743 |
| 3m66 | 0.752083 | 0.719689 |
| 3mbr | 0.700058 | 0.726271 |
| 3mvs | 0.63209 | 0.731889 |
| 3ne0 | 0.614757 | 0.719187 |
| 3nir | 0.832858 | 0.677223 |
| 3onh | 0.842973 | 0.729113 |
| 3osx | 0.752386 | 0.719845 |
| 3piw | 0.7803 | 0.722137 |
| 3puc | 0.699228 | 0.670211 |
| 3qx1 | 0.881822 | 0.795356 |
| 3rjp | 0.752206 | 0.719875 |
| 3rkg | 0.810524 | 0.728506 |
| 3rt2 | 0.891228 | 0.726741 |
| 3rx9 | 0.839569 | 0.731215 |
| 3t3l | 0.749189 | 0.717825 |
| 3t7l | 1.001859 | 0.708338 |
| 3tn2 | 0.67482 | 0.722744 |
| 3tow | 0.636048 | 0.672099 |
| 3tyt | 0.817389 | 0.714778 |
| 3us6 | 0.776574 | 0.719042 |
| 3v46 | 0.848864 | 0.671655 |
| 3vmn | 0.736626 | 0.720212 |
| 3vmv | 0.523807 | 0.728318 |
| 3vor | 0.789987 | 0.673882 |
| 3zbd | 0.722187 | 0.79562 |
| 3zsu | 0.589619 | 0.71718 |
| 3zzo | 0.785419 | 0.714968 |
| 3zzp | 0.498375 | 0.719334 |
| 4a02 | 0.681566 | 0.721748 |
| 4a4j | 0.836937 | 0.71945 |
| 4a9v | 0.629524 | 0.69467 |
| 4abl | 0.748028 | 0.716022 |
| 4acj | 0.703594 | 0.695162 |
| 4ann | 0.881921 | 0.721423 |
| 4b89 | 0.704404 | 0.722036 |
| 4b9g | 0.871578 | 0.745455 |
| 4d8b | 0.720093 | 0.669533 |
| 4dvc | 0.796113 | 0.674352 |
| 4e40 | 0.679079 | 0.713182 |
| 4eb0 | 0.687233 | 0.721793 |
| 4esm | 0.574928 | 0.728669 |
| 4f1v | 0.676015 | 0.677826 |
| 4f2f | 0.503634 | 0.724727 |
| 4ftf | 0.803677 | 0.717525 |
| 4g3o | 0.720667 | 0.72931 |
| 4ga2 | 0.83672 | 0.668947 |
| 4gc3 | 0.710273 | 0.725398 |
| 4gco | 0.816728 | 0.70554 |
| 4gei | 0.834802 | 0.726034 |
| 4gmq | 0.585199 | 0.714674 |
| 4gzc | 0.786858 | 0.719938 |
| 4h4n | 0.862857 | 0.710759 |
| 4he6 | 0.694133 | 0.72214 |
| 4hu2 | 0.639704 | 0.724403 |
| 4i6x | 0.795733 | 0.717483 |
| 4iej | 0.821463 | 0.715515 |
| 4il7 | 0.747073 | 0.733556 |
